# Anti-modularization for both high robustness and efficiency including the optimal case

**DOI:** 10.1371/journal.pone.0301269

**Published:** 2024-03-28

**Authors:** Jaeho Kim, Yukio Hayashi

**Affiliations:** Divison of Transdisciplinary Sciences, Japan Advanced Institute of Science and Technology, Nomi, Ishikawa, Japan; Northeastern University, UNITED STATES

## Abstract

Although robustness of connectivity and modular structures in networks have been attracted much attentions in complex networks, most researches have focused on those two features in Erdos-Renyi random graphs and Scale-Free networks whose degree distributions follow Poisson and power-law, respectively. This paper investigates the effect of modularity on robustness in a modular *d*-regular graphs. Our results reveal that high modularity reduces the robustness even from the optimal robustness of a random *d*-regular graph in the pure effect of degree distributions. Moreover, we find that a low modular *d*-regular graph exhibits small-world property that average path length is *O*(*logN*). These results indicate that low modularity on modular structures leads to coexistence of both high robustness and efficiency of paths.

## Introduction

Energy, transportation, and communication systems provide essential services for supporting human activity and society. However, in these network systems, there is a common topological structure called Scale-Free (SF), which has the extreme vulnerability against malicious attacks to hubs [1]. Therefore, constructing more robust networks is one of the important issues in complex networks. Recently, it has been revealed that enhancing loops is crucial for constructing a robust network in supporting from the asymptotical equivalence of network dismantling and decycling problems when the second moment of degree is not divergent [2]. Here, the dismantling problem is to find the minimum set of nodes which removal makes a network fragmented into at most a given size, while the decycling problem is to find the minimum set of nodes which are necessary to form loops. When all loops are removed from a network, the network becomes a tree which is easily fragmented by any articular-node removals. Thus, enhancing loops is important to make a network hard to become a tree. Actually, several rewiring methods [3] based on enhancing loops generate robust networks with a common phenomena of decreasing the gap between the maximum and minimum degrees. In other words, the network is more robust as the gap becomes smaller. In the extreme case, it is suggested that a random *d*-regular graph with zero gap has the optimal robustness in the pure effect of degree distributions [4, 5]. Regular graphs have been so far studied mainly for not robustness but spectral analysis [6] or graph theory, while there are huge researches [7, 8] for robustness in Erdos-renyi (ER) random graphs and SF networks. Therefore, regular graphs become a blind spot in investigating the robustness of connectivity.

On the other hand, a modular structure is also an important issue in complex networks, because many real-world networks have modular structures. For example, in social networks, modules are corresponding to communities or groups with shared interests or backgrounds. If a network has high modularity, nodes in a same module are densely connected to each other, whereas nodes in different modules are sparsely connected.

Recently, it has been shown that [9, 10] ER random graphs and SF networks with modular structures become weaker against attacks than them without modular structures. Here, the modularity of networks is controlled by rewiring links. Moreover, Module-Based (MB) attacks which are targeting interconnected nodes with high betweenness centrality are highly destructive to modular networks [11]. Thus, we predict that random *d*-regular graphs with modules become vulnerable against MB attacks, even the original graphs have the optimal robustness [4, 5]. The modularity of the modular network is controlled by rewiring inspired from [9, 10] with preserving degree distributions. In addition, we show that *d*-regular graphs with low modularity have both high robustness of connectivity and efficiency of paths.

This paper is organized as follows. First, we introduce a modular network of *d*-regular graphs and an anti-modularization that is rewiring links to decrease the modularity. Second, we show that rewired networks on anti-modularization have high robustness of connectivity and efficiency of paths. Third, we modify the conventional modularization [10] to maintain *d*-regular graphs and show that modified modularization has the same results with our anti-modularization. Finally, we summarize the obtained results.

## Control of modularity

**Fig 1:**
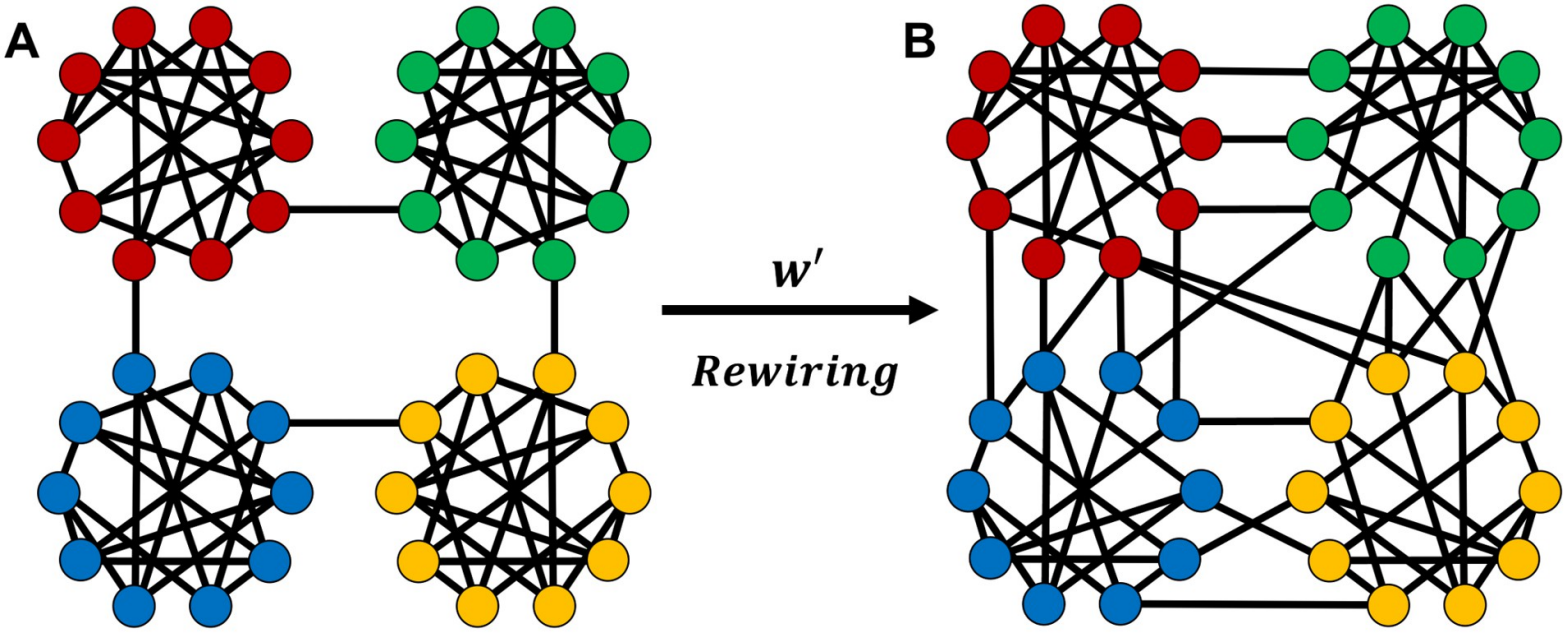
Configuration of a modular network before/after rewirings. (A) Initial configuration of strongly modular network. (B) Rewired modular network to increase the robustness of connectivity. Node colors indicate modules.

We consider a modular network that consists of random *d*-regular graphs. Each of random *d*-regular graphs corresponds to a module. As shown in Fig 1A, *m*_*o*_ modules with size *N*_*m*_ are initially connected as a ring. Here, the total number of nodes and links are constant *N* = *N*_*m*_ × *m*_*o*_ and *M* = (*d* × *N*)/2 in the network. The initial configuration of modular network has considerably high modularity, because a ring structure maintains the connectivity of the entire network by the minimum number of inter-module links (inter-links) and the maximum number of intra-module links (intra-links). However, this network is extremely vulnerable by removing target nodes which are connecting between modules. As a special case, when each module is set as a clique, such network is called caveman graph [12]. For a caveman graph, small-world (SW) property with the average path length of *O*(*logN*) emerges by rewiring from a few intra-links to inter-links. We discuss not only the robustness but also the efficiency with SW property in modular networks of *d*-regular graphs.

To control the modularity of network, intra-links are rewired to inter-links on anti-modularization as shown in Fig 1B. This is regarded as the inverse process of the conventional modularization [10], although it tends to not make a ring as mentioned later. Table 1 shows the initial number of intra- and inter-links. By rewirings, the number of intra-links is only decreasing on anti-modularization, because the sum of intra- and inter-links is constant *M*. In contrast, the number of inter-links is only decreasing on modularization [10]. With a rewiring rate *w*′, intra-links are randomly rewired to inter-links on anti-modularization as shown in Fig 1(B), while with a rewiring rate *w*, inter-links are randomly rewired to intra-links on modularization [10]. Moreover, the relation of *w*′ and *w* is derived from the following equation

$$\begin{array}{c} m_{o}+w^{\prime }\left(M-m_{o}\right)w^{\prime }=\left(1-w\right)\left(M-M/m_{o}\right), \end{array}$$ (1)

whose left- and right-hand sides are the existing number of inter-links on anti-modularization and the remaining number of them on modularization [10] after both rewirings. Then, we have

$$\begin{array}{c} w^{\prime }=1-\frac{1+w(m_{o}-1)}{m_{o}\left(M-m_{o}\right)}M. \end{array}$$ (2)

**Table 1:** Initial numbers of intra- and inter- links.

	Intra-links	rewirings	Inter-links
anti-modularization	*M* − *m*_*o*_	$\underset{\to }{w^{\prime }}$	*m*_*o*_
modularization [10]	*M*/*m*_*o*_	$\underset{\leftarrow }{w}$	*M* − *M*/*m*_*o*_

The detail process of anti-modularization is summarized as follows. Through the process, the degree distributions and whole connectivity of rewired networks are maintained, while they are not on the conventional modularization [10]. We will compare such differences in the next section.

Step 1 At first, remove an intra-link randomly in a module. Then, randomly select one end-node of the removed link.
Step 2 Remove another intra-link randomly in a different module from that in Step1. Then, randomly select one end-node from the removed link.
Step 3 Create a new inter-link between two selected nodes by rewiring.
Step 4 Remove another intra-link randomly in a different module from that in Step 2 or the previous Step 4. Then, randomly select one end-node from the removed link.
Step 5 To preserve the degree at a node, the unselected end-node in Step 2 or the previous Step 4 is selected again. Create a new inter-link between two selected nodes by rewiring.
Step 6 Steps 4 and 5 are repeated until *w*′(*M* − *m*_*o*_) inter-links are created for a given 0 < *w*′ < 1. If connected components or nodes are isolated by rewiring, select other end-node or intra-link again. At the end of repeated process, the last inter-link is connected to the node which is unselected in Step 1.

To investigate the robustness of connectivity, the following three types of attacks RF, IB, and MB are considered. In RF (Random Failures), nodes are randomly selected for removal. In IB (Initial Betweenness) attacks, nodes are selected in decreasing order of betweenness centrality. In MB (Module-Based) attacks [11], nodes are basically selected in decreasing order of betweenness centrality, however the nodes belong to the Largest Connected Component (LCC) and end-nodes of inter-links have the order of priority. MB and IB attacks are highly distructive especially for modular networks [11], while RF gives well-known typical damages and is considered to compare the robustness with them.

## Coexistance of robustness and efficiency

We investigate the robustness of connectivity and efficiency of paths in rewired networks on anti-modularization. In addition, we compare the robustness in rewired networks on both anti-modularization and modified modularization to maintain degree distribution, and show that the results for both modularization are almost coincidence. Here, the degree and the size of modular network are *d* = 4, 9, 19 and *N* = 10^4^. Since *N* is constant, the size of each module *N*_*m*_ is also constant for a given number *m*_*o*_ of modules which is a control parameter. When *m*_*o*_ is maximum, the module becomes a clique *K*_*d*+1_ in a caveman graph [12]. Modularity *Q* and following eight measures are investigated and are averaged over 100 realizations for the networks in varying a rewiring rate *w*′.

- Modularity [13] $Q=\frac{1}{2M}\sum_{i,j}\left(A_{ij}-\frac{k_{i}k_{j}}{2M}\right)\delta_{i,j}$, where *A* is adjacency matrix, *k*_*i*_ is degree of node *i*, and *δ*_*i*,*j*_ is 1 if nodes *i* and *j* belong to a same module or 0 otherwirse.
- Ratio *S*^1*st*^(*q*)/*N* of the 1st LCC size, where *S*^1*st*^(*q*) denotes the number of nodes in the 1st LCC after attacks to *qN* nodes. 0 < *q* ≤ 1 is a fraction of attacks.
- Robustness
  1. Robustnes index [14] $R=\frac{1}{N}\sum_{q=\frac{1}{N}}^{1}\frac{S(q)}{N}$ after attacks to *qN* nodes. The summation means $q=\frac{1}{N},\frac{2}{N},...\frac{N-1}{N}$, and $\frac{N}{N}=1.$
    Except for *R*, there are other following measures of robustness [15] in the viewpoints of shortest paths and graph spectrum.
  2. Reciprocal of network efficiency $H=\frac{1}{\frac{2}{N(N-1)}\sum_{i\in V}\sum_{j\in V,i\neq j}\frac{1}{d_{ij}}}$, where *d*_*ij*_ denotes the shortest path length between nodes *i* and *j*. *H* is called harmonic mean.
  3. Average path length $L=\frac{1}{N(N-1)}\sum_{i,j}d_{ij}$, where *d*_*ij*_ denotes the shortest path length between nodes *i* and *j*. *L* is called arithmetic mean.
  4. Diameter *D* = *max*{*d*_*ij*_}, where *d*_*ij*_ is the shortest path length between node *i* and *j*. Small values of *H*, *L* and *D* mean that a network is robust.
  5. Average betweenness centrality $b=\frac{1}{N}\sum_{k\in V}\sum_{i\in V}\sum_{j\in V,i\neq j\neq k}\frac{n_{ij}\left(k\right)}{n_{ij}}$, where *n*_*ij*_(*k*) is the number of shortest paths between node *i* and *j* through node *k*. A small value of *b* indicates that lots of nodes are connected by the shortest path without relying on specific nodes like hubs.
  6. Spectral gap λ_*d*_ = λ_1_ − λ_2_, which is the difference between the largest and second largest eigenvalues λ_1_ and λ_2_ of the adjacency matrix of a network. Since a small value of λ_*d*_ is related to network bottlenecks and bridges, a larger value of λ_*d*_ indicates better robustness.
  7. Algebraic connectivity *μ*_2_, which is the second smallest eigenvalue of the Laplacian matrix of a network. A larger value of *μ*_2_ means more rapid diffusion.

### Increasing robustness by anti-modularization

**Fig 2:**
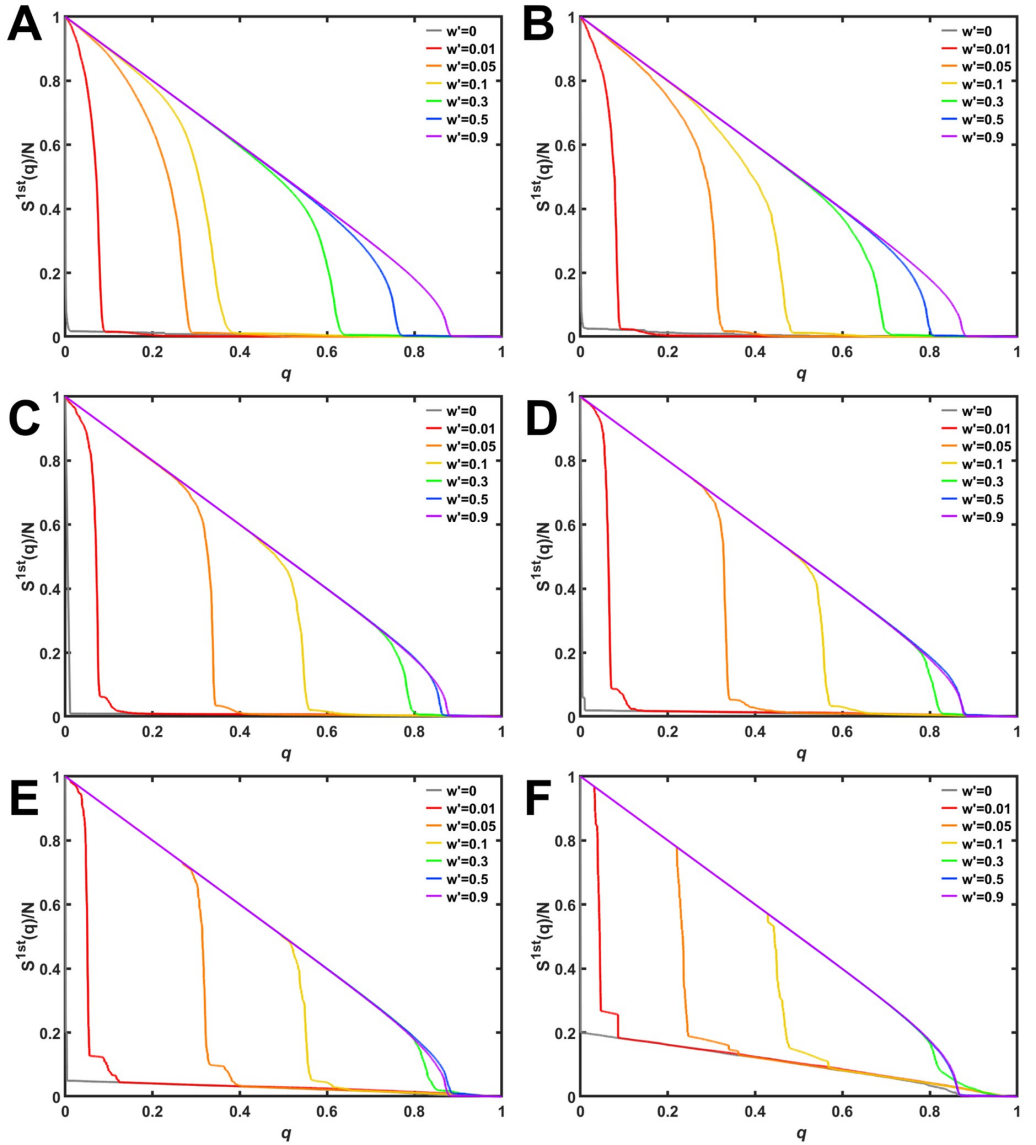
The 1st LCC size against MB attacks in rewired networks with *d* = 9 and the number *m*_*o*_ of modules. (A) *m*_*o*_ = 1000, (B) *m*_*o*_ = 500, (C) *m*_*o*_ = 100, (D) *m*_*o*_ = 50, (E) *m*_*o*_ = 20, and (F) *m*_*o*_ = 5. Color lines represent the rewiring rates *w*′ on anti-modularization.

Mainly, we explain the results for *d* = 9. See figures from S3 to S8 Figs as similar results obtained for *d* = 4 and *d* = 19. We investigate the ratio *S*^1*st*^(*q*)/*N* of the 1st LCC size against MB attacks in rewired networks on anti-modularization. Fig 2A–2F are corresponding to 9-regular graphs with *m*_*o*_ = 1000, 500, 100, 50, 20, and 5, respectively. As shown in Fig 2A, the curve for *w*′ = 0 (gray line) decreases very rapidly because of initial ring structure. However, as the rewiring rate *w*′ increases on anti-modularization, the curves are shifting to right. At *w*′ = 0.9, the curve is approaching purple lines for non-modular random *d*-regular graphs. Such curve-shifting are also observed in the results for different size of modules *m*_*o*_ = 500, 100, 50, 20, and 5 in each of Fig 2B–2F. In comparing with same color lines in Fig 2A–2E, curves are more shifting to right as smaller *m*_*o*_.

On the other hand, in Fig 2A–2F, curves for *w*′ ≥ 0.5 (blue and purple lines) are almost identical. Moreover, as shown in Fig 2F, for *w*′ = 0 and 0.1 (gray and red lines), the curves rapidly decrease to *S*^1*st*^(*q*)/*N* ≈ *N*_*m*_/*N* = 2000/10000 = 0.2 at first. Then, the curves gradually decrease from around 0.2. Note that MB attacks destroy a network in two steps. First, MB attacks are targeting the end-nodes of inter-links, and divide into several isolated modules. Next, each of isolated modules is collapsed gradually. See S1 and S2 Figs in cases of IB attacks and RF.

Table 2 shows a critical fraction *q*_*c*_ denoted by $q_{c}^{MB}$, $q_{c}^{IB}$, and $q_{c}^{RF}$ at the maximum size of the 2nd LCC against MB, IB attacks and RF in rewired networks with *d* = 9 and *m*_*o*_ = 200. Since the 1st LCC is fragmented at the critical fraction *q*_*c*_, the whole connectivity is broken considerably. Values of $q_{c}^{MB}$, $q_{c}^{IB}$, and $q_{c}^{RF}$ increase as larger *w*′. Especially, $q_{c}^{MB}$, $q_{c}^{IB}$ are rapidly increasing between the case of *w*′ = 0.1 and *w*′ = 0.3. When *w*′ ≤ 0.3, $q_{c}^{RF}$ is higher than $q_{c}^{MB}$ and $q_{c}^{IB}$. However, when *w*′ ≥ 0.5, $q_{c}^{RF}$ becomes lower than $q_{c}^{MB}$ and $q_{c}^{IB}$. In particular, for *w*′ = 0.9, $q_{c}^{IB}$ is approaching 0.875, which is the percolation threshold 1 − 1/(*d* − 1) = 1 − 0.125 for *d* = 9 in random *d*-regular graphs [4]. From Table 2, we find that random *d*-regular graphs with low modularity (*w*′ ≥ 0.5) have stronger robustness with high *q*_*c*_ against selected IB attacks than that against unintended RF. This phenomena is unusual in ER random graphs or SF networks [16]. As a considerable reason, IB attacks remove only the core part which is frequently passed by the shortest paths, while the remaining nodes maintain the connectivity on the peripheral in *d*-regular graphs.

**Table 2:** Values of the critical fraction *q*_*c*_ against three types of attacks in rewired networks with *d* = 9 and *m*_*o*_ = 200.

	w’=0.01	w’=0.05	w’=0.1	w’=0.3	w’=0.5	w’=0.9
MB	0.075	0.340	0.546	0.786	0.858	0.874
IB	0.092	0.353	0.559	0.803	0.886	0.911
RF	0.592	0.775	0.809	0.848	0.850	0.861

**Fig 3:**
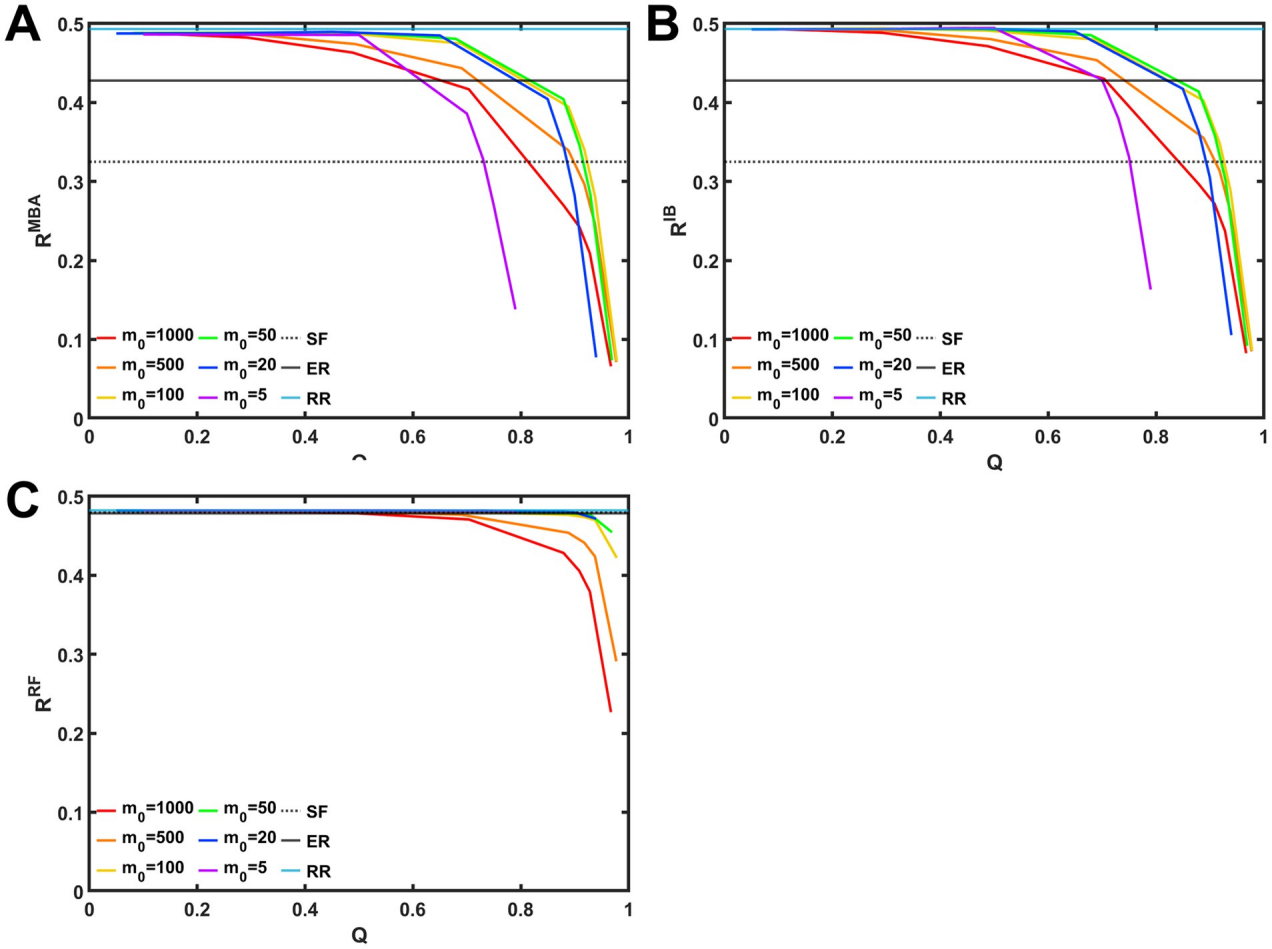
Relation between modularity *Q* and robustness index *R* against three types of attacks. (A) *R*_*MB*_ against MB, (B) *R*_*IB*_ against IB, and (C) *R*_*RF*_ against RF. Color lines represent the results for the number *m*_*o*_ of modules. Black solid, dotted, and cyan lines represent the robustness after these attacks in non-modular ER, SF networks and random *d*-regular graphs.

We note that the rewired networks for high *w*′ on anti-modularization have low modularity. Fig 3 shows that robustness index *R* against each of three types of attacks is monotonically decreasing function of modularity *Q* even for varying *m*_*o*_ shown by color lines. In Fig 3A and 3B, when *Q* < 0.2, all colored curves are approaching cyan lines, which indicates the robustness index *R*_*IB*_ against IB attacks for non-modular random 9-regular graphs. All colored curves in Fig 3C are also approaching cyan lines when *Q* < 0.5. In addition, there are two behaviors among curves in Fig 3A and 3B. One is that, from *m*_*o*_ = 1000 to *m*_*o*_ = 50, red curves are shifting to green curves. The other is that, from *m*_*o*_ = 50 to *m*_*o*_ = 5, green curves are shifting to purple curves. However, these behaviors are not observed in in Fig 3C. The reasons for such behaviors are unknown in the current stage, and will be investigated in future works.

We should remark that *R*_*MB*_, *R*_*IB*_ and *R*_*RF*_ in rewired networks with high *Q* become lower than those in non-modular ER networks (black line) and SF networks known as the extremely vulnerable structure (black dotted line). This means that modular random *d*-regular graphs become more vulnerable than SF networks by increasing the modularity, even if non-modular random *d*-regular graphs have the optimal robustness against malicious attacks [4, 5]. Note that robustness against IB and Initial Degree (ID) attacks also decrease as increasing the modualarity *Q* in SF networks and real-world networks [10].

**Fig 4:**
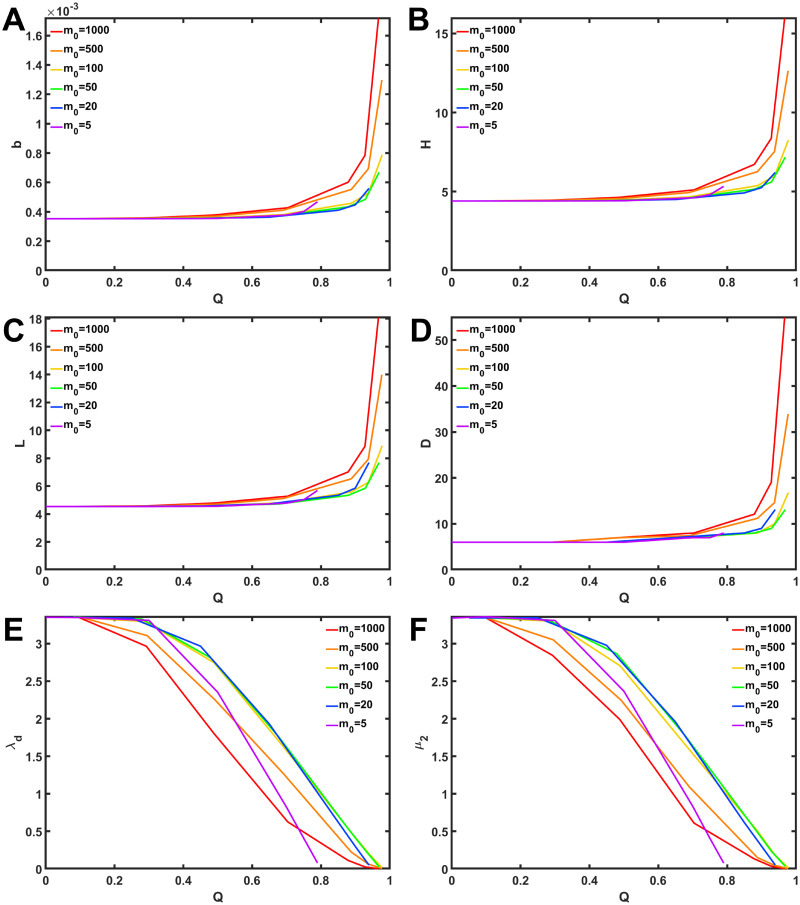
Relation between modularity *Q* and six measures of robustness in rewired networks with *d* = 9. (A) Average betweenness centrality, (B) reciprocal of network efficiency, (C) average path length, (D) diameter, (E) spectral gap, (F) algebraic connectivity. Color lines represent the results for the numbers *m*_*o*_ of modules.

As shown in Fig 4A–4F, we investigate other measures of robustness: average betweenness centrality, the reciprocal of network efficiency, average path length, diameter, spectral gap, and algebraic connectivity. Consequently, when modularity *Q* increase, the values of average betweenness centrality, the reciprocal of network efficiency, average path length, and diameter also increase (Fig 4A–4D), while the values of spectral gap and algebraic connectivity decrease (Fig 4E and 4F). These results indicate that networks with low modularity have good robustness. Similar results are obtained for other networks with varying *d* as shown in S9 and S10 Figs.

### Emerging SW property by anti-modularization

Moreover, we have investigated efficiency of paths in two ways. First, as similar to the result of SW property for a caveman graph [12], we show that SW property emerges in regular graphs. Then, we compare the numerical values of average path length with newly proposed estimation [17].

**Fig 5:**
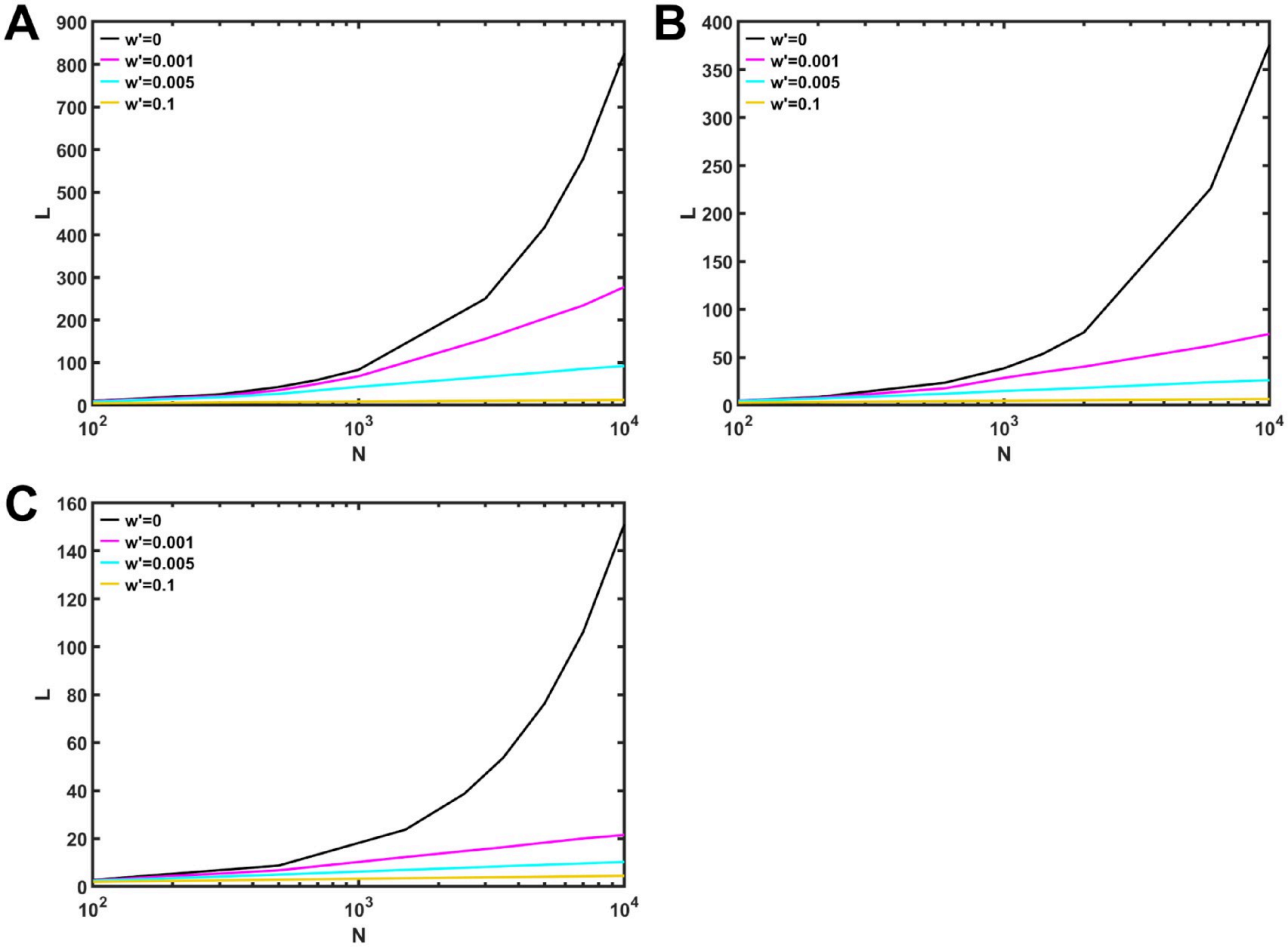
Average path length *L* in rewired networks for varying the network size *N*. Rewired networks with (A) *d* = 4, *N*_*m*_ = 10, (B) *d* = 9, *N*_*m*_ = 20, and (C) *d* = 19, *N*_*m*_ = 50. Color lines represent a rewiring rate *w*′ on anti-modularization.

Fig 5A shows the average path length *L* as function of network size *N*, when *d* = 4 and *N*_*m*_ = 10 are fixed. In a ring structure (when *w*′ = 0), *L* is *O*(*N*) (black line). However, even only rewiring a few intra-links, *L* becomes *O*(*logN*) for *w*′ = 0.1 (yellow line). This indicates that a network has SW property [12]. Similar results are obtained for other networks in Fig 5B with *d* = 9, *N*_*m*_ = 20 and in Fig 5C with *d* = 19, *N*_*m*_ = 50. It is common that *L* is *O*(*logN*) in these networks for *w*′ = 0.1. In addition, figures from S11 to S13 Figs show that SW property emerges in rewired networks even for varying *d* and *N*_*m*_. Remember the result in caveman graphs [12], which consist of cliques initially connected as a ring. Our obtained results include the robustness for the caveman graphs in Fig 2A, because cliques are the most densely case of regular graphs.

**Fig 6:**
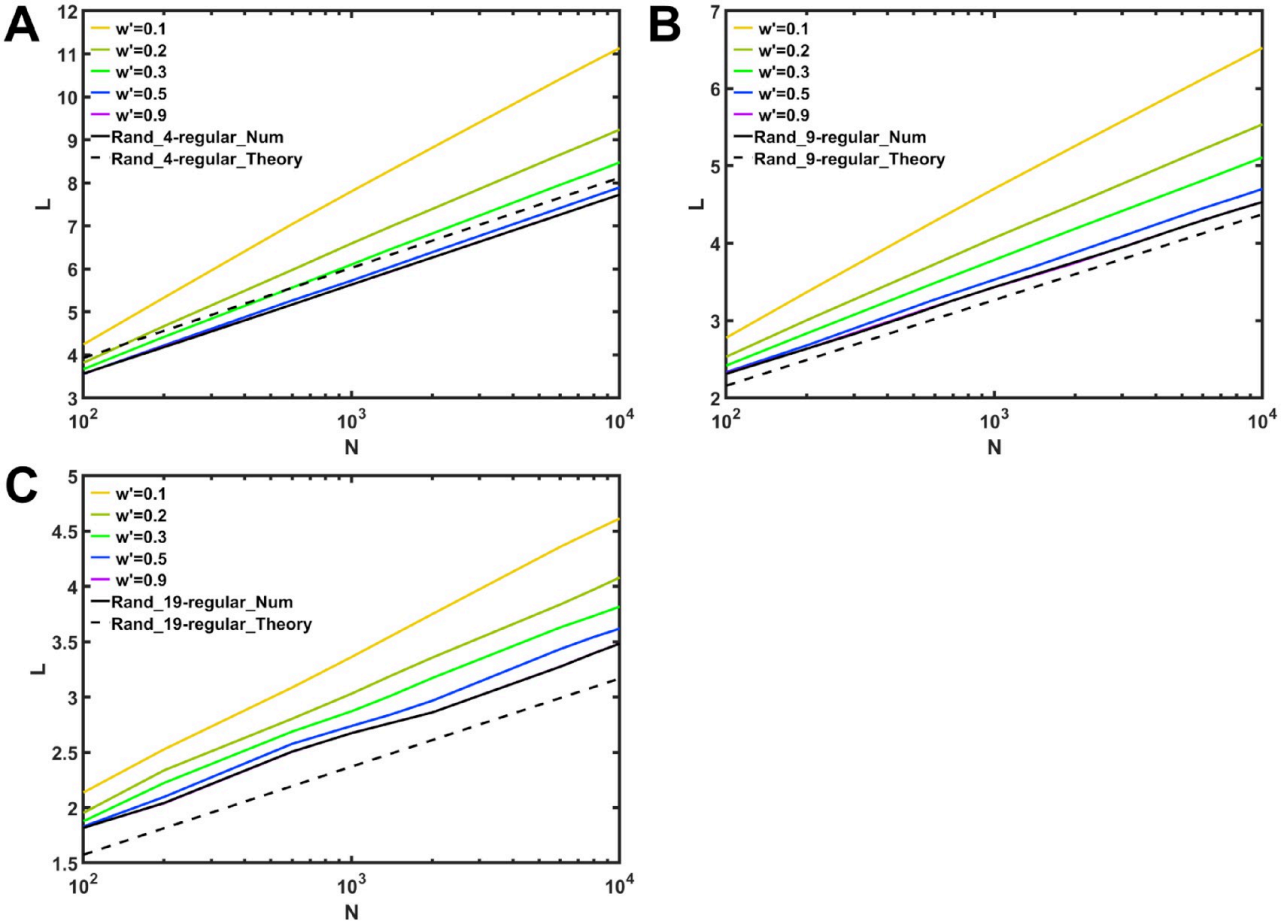
Disparities of average path length *L* between the values for rewired modular networks and theoretical value. Rewired networks with (A) *d* = 4, *N*_*m*_ = 20, (B) *d* = 9, *N*_*m*_ = 20, and (C) *d* = 19, *N*_*m*_ = 20. Color lines represent a rewiring rate *w*′ on anti-modularization. Black solid and dashed lines indicate numerical and theoretical values for random *d*-regular graphs without modular structures.

In general for a sparse network with any degree distribution, the average path length

$$\begin{array}{c} L\approx \frac{log(N/\langle k\rangle )}{log(\left(\langle k^{2}\rangle -\langle k\rangle \right)/\langle k\rangle )}+1 \end{array}$$

is derived through the analysis of generating functions [18]. From 〈*k*〉 = *d* and 〈*k*^2^〉 = *d*^2^ for a *d*-regular graph, the estimation

$$\begin{array}{c} L\approx \frac{log(N/d)}{log(d-1)}+1 \end{array}$$ (2)

is obtained approximately as SW property. Thus, we compare Eq (2) with our results for the modular networks after rewirings. Fig 6 shows that the color lines of *O*(*logN*) with slightly different slops approach black solid line for random *d*-regular graphs. In particular, purple line for the case with *w*′ = 0.9 almost coincides with black solid line, while there is a small gap between black solid and dashed lines. Note that dashed line corresponds to Eq (2). However, it has been pointed out that there exist disparities between numerically obtained average path length and more rigorous estimation than Eq (2) for random regular graphs, when they are dense [17]. Our study for *d* = 4, 9 and 19 ≪ *N* is classified as sparse graphs, the derivation of more rigorous estimation is intractable for the modular networks. Therefore, it will be a future work to investigate the existing such disparities.

### Comparing with modified modularization as the inverse process of anti-modularization

**Fig 7:**
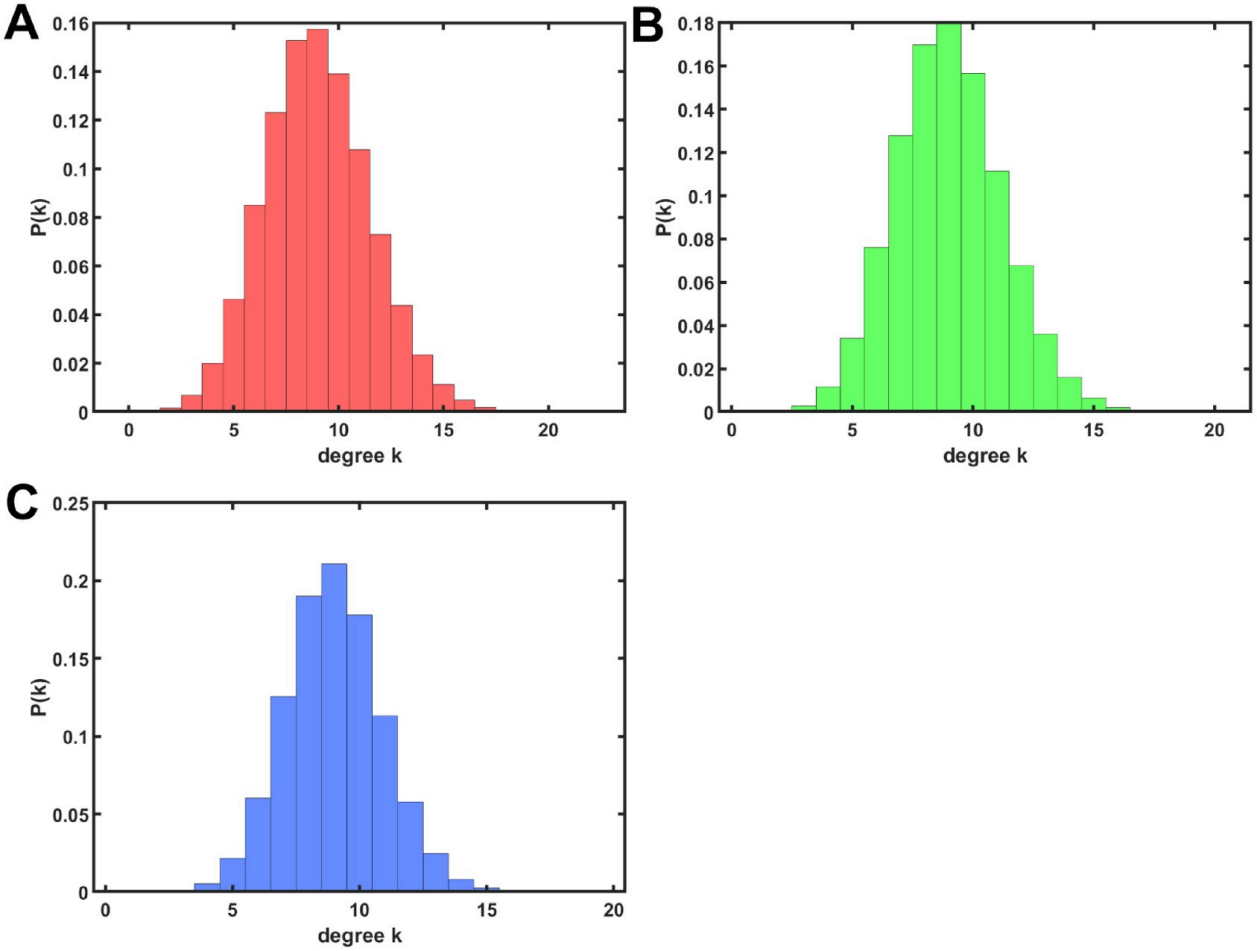
Degree distribution in rewired networks with *d* = 9 and *m*_*o*_ = 100. For rewiring rates (A) *w* = 0.988, (B) *w* = 0.695, and (C) *w* = 0.494. The values of *w* are corresponding to *w*′ = 0.01, 0.3, 0.5 from Eq (1).

Since the conventional modularization [10] does not maintain a degree distribution and whole connectivity, we propose modified process of it. Fig 7A–7C show that the degree distributions have widths in rewired networks with *d* = 9 and *m*_*o*_ = 100 on the conventional modularization [10]. We confirm that degree distributions also have widths in rewired networks for varying *d* and *m*_*o*_. This means that rewired networks are no longer random *d*-regular graphs on the conventional modularization [10].

**Fig 8:**
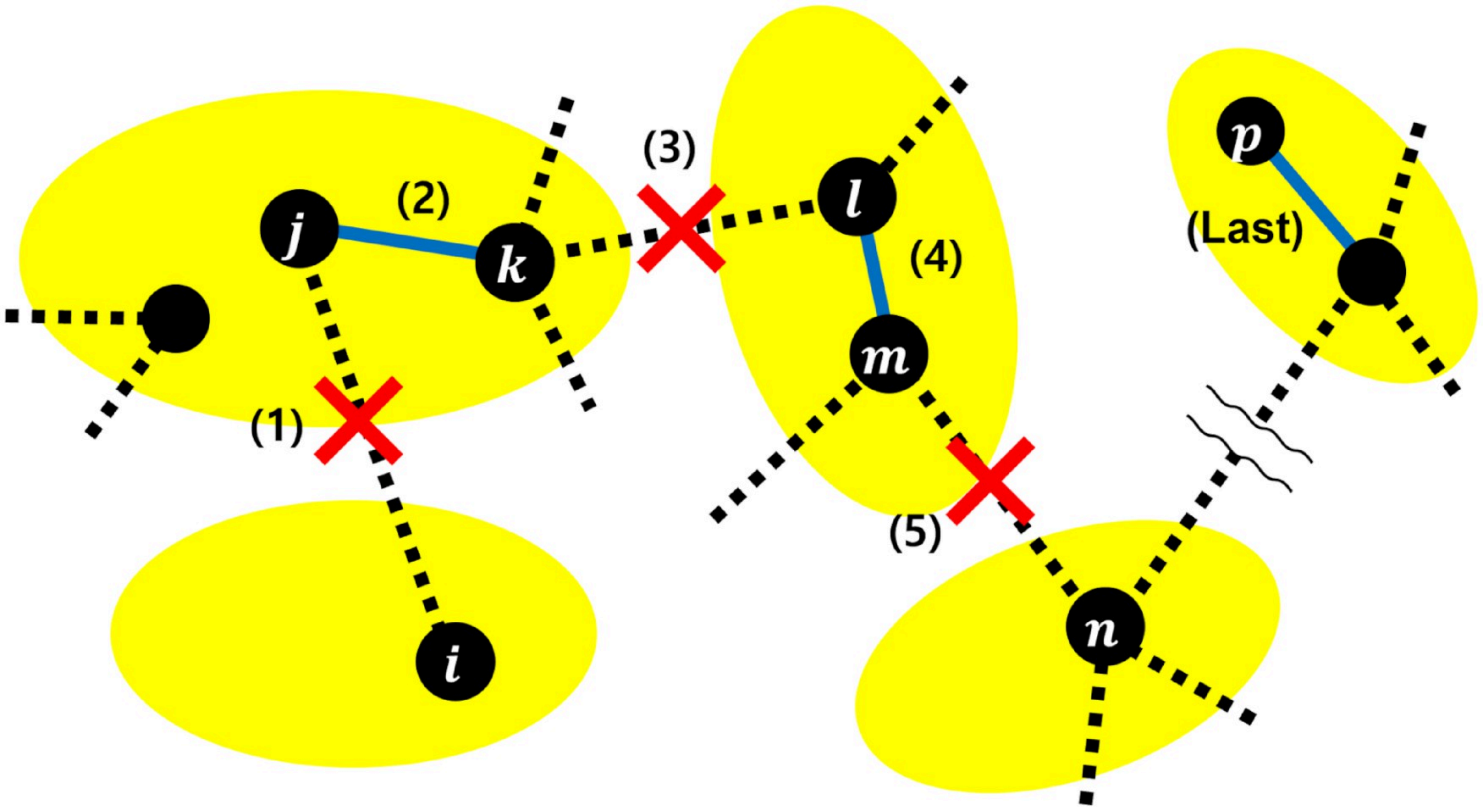
Process of the modified modularization. The numbers in brackets denote a sequence of the process. Yellow circles represent modules. Black dotted and Blue solid lines are inter- and intra-links by rewirings, respectively.

Thus, we modify the process of modularization [10] to maintain connected *d*-regular graphs. The basic idea is similar to our anti-modularization. Fig 8 illustrates the modified process. Initially, module numbers 1, 2, …, *m*_*o*_ are assigned to nodes randomly, while a size of each module *N*_*m*_ is constant. (1) An inter-link (*i*, *j*) is randomly removed. Then, one end-node *j* of the removed link is selected randomly. (2) A node *k* is randomly selected, which belongs to a same module with node *j* and does not connected to node *j*. Then, a new intra-link is created between nodes *j* and *k*. (3) To preserve a node degree, an inter-link (*k*, *l*) is randomly removed. (4) Select node *m* in a same module with node *l* randomly. Then, create a new intra-link between nodes *l* and *m*. (5) An inter-link (*m*, *n*) is randomly removed. After (5), node *n* is the next target for rewiring. Such process is repeated until *w*(*M* − *M*/*m*_*o*_) intra-links are created without isolation of connected components and nodes. However, at the end of repeated process, the degree of first selected node *i* is decreased by one at (1), while the degree of last selected *p* is increased by one.

**Fig 9:**
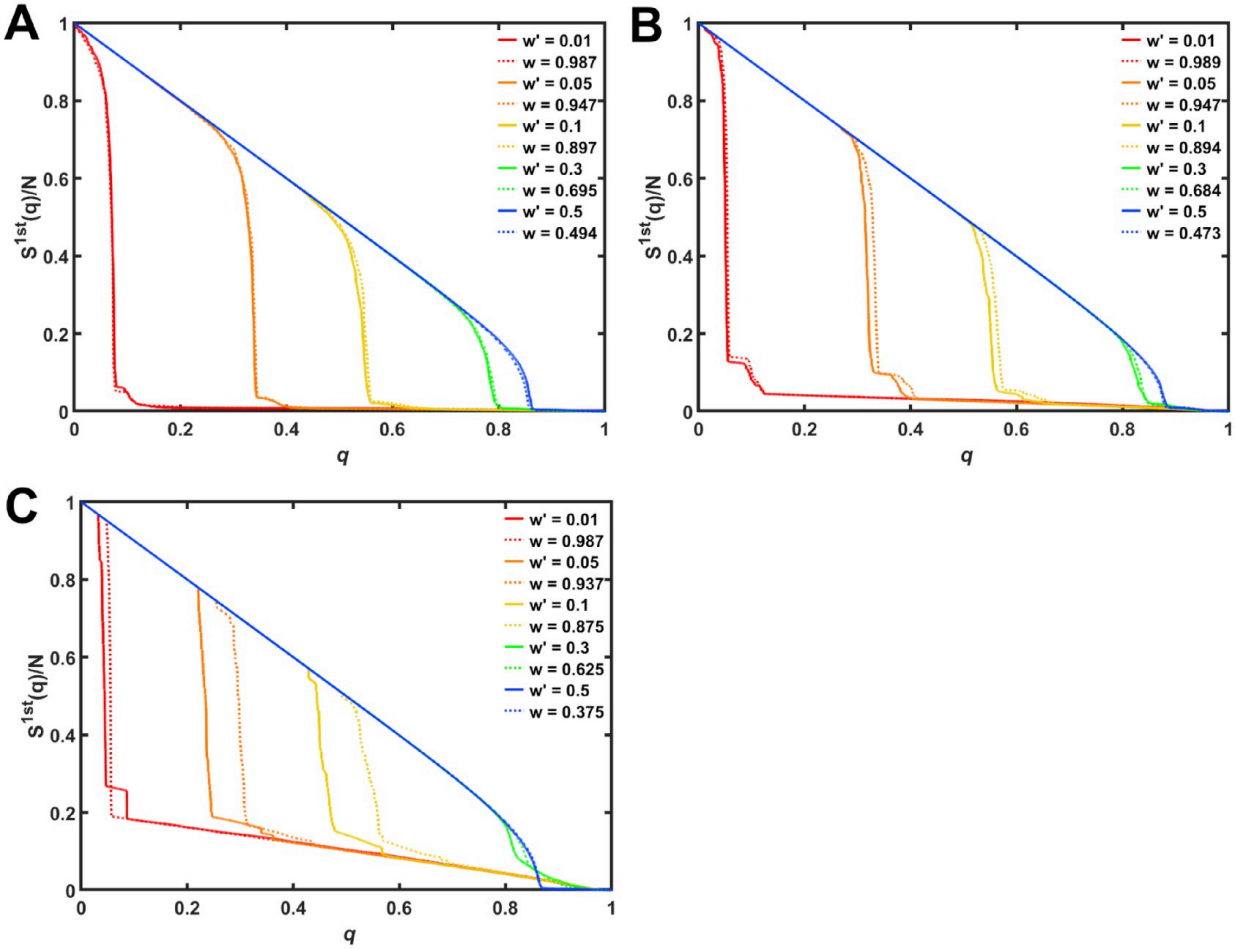
Comparing the robustness against MB attacks in rewired networks on the anti-modularization and modified modularization. The 1st LCC size against MB attacks in rewired networks with *d* = 9 and number *m*_*o*_ of modules. (A) *m*_*o*_ = 100, (B) *m*_*o*_ = 20, and (C) *m*_*o*_ = 5. Solid and dotted color lines represent the results by rewiring with *w*′ on anti-modularization and *w* on modified modularization.

As shown in Fig 9A for the robustness, curves of each colors on modified modularization (dotted lines) and anti-modularization (solid lines) are almost identical in rewired networks with *m*_*o*_ = 100. However, as smaller number *m*_*o*_ of modules, the gaps between solid and dotted lines are larger. Especially, in rewired networks with *m*_*o*_ = 5, such gaps are remarkable for orange and yellow lines in Fig 9C. Red lines in Fig 9A–9C are almost identical, although rewired networks for high rewiring rate *w* on modified modularization tend to not have a ring structure. See S15 and S16 Figs in cases of IB attacks and RF.

## Conclusion

We have numerically investigated the robustness of connectivity in modular *d*-regular graphs which have the optimal tolerance in the non-modular random case. The robustness against MB, IB attacks and RF increases, when more intra-links are rewiring on anti-modularization. In particular, our results have shown that the robustness decreases rapidly against MB attacks than IB attacks as the modularity increases. When the modularity *Q* is above 0.8, the robustness of modular *d*-regular graphs becomes lower than the robustness of very fragile SF networks. Moreover, we have shown that both high robustness and efficiency coexist in rewired networks on anti-modularization. In fact, like caveman graphs [12], modular *d*-regular graphs exhibit SW property by rewiring only a few intra-links for *w*′ = 0.1, whereas the numerical values of *L* for modular networks are slightly different from the well-known theoretical values [18] for random regular graphs. At *w*′ = 0.1, modular *d*-regular graphs still have low robustness. Thus, in order to be both high robust and efficient, more intra-links at least with 0.1 < *w*′ < 0.3 are needed in modular *d*-regular graphs.

Unlike our results for regular graphs with *N* = 10^4^, the previous study [9] shows that there exists a critical number $m_{o}^{*}$ of modules below which ER random graphs are completely broken into modules by attacks on interconnected nodes. In particular, for ER random graphs with *N* = 6 × 10^5^, discontinuous jump of the 1st LCC size occurs for a critical number m* of modules. Although the study [9] also considers a modular network that consist of *m*_*o*_ modules, it additionally defines the ratio *α* between the probabilities for an intra- and inter-links. With constant average degree 〈*k*〉 and ratio *α*, the percolation threshold is shown as a function of *m*_*o*_ modules. However, in varying *m*_*o*_ for constant *N* = *m*_*o*_ × *N*_*m*_ and *M* = *N* × *d*/2, if *α* is fixed, rewiring rate *w* is also changed simultaneously because of $\alpha =\frac{m_{o}}{1-w}-\left(m_{o}-1\right)$ [10]. Hence there exists such differences, it is unknown whether or not the discontinuous jump also appears in our case for very larger *N*. Thus, it is required to compare the results in this point.

On the other hand, we find that modular *d*-regular graphs with low modularity are more robust against selected IB attacks than against unintended RF. We also confirm that the robustness of rewired networks on modified modularization is almost identical to that on the anti-modularization which is the inverse process of the modified one. However, there still remains some unclear reasons for our results. For example, as shown in S4A Fig, curves of *R*_*IB*_ with *w*′ = 0.01 and 0.1 have inverse relationship. In addition, in Fig 2F, S3F and S6F Figs, some curves of the 1st LCC size with *w*′ = 0.9 and *w*′ = 0.1 are crossing at high attack rates *q* ≃ 0.85, 0.7, and 0.92 of node removals. Thus, further investigations are necessary for such phenomena.

Although huge computation times are required, it will be future works to investigate the robustness of connectivity against various attacks: edge attacks [19], attacks based on other node centrality [20], CoreHD [21], collective influence [22], belief propagation [23], or spatial localized attacks [24]. Moreover, expanding our analytical framework may be meaningful as another direction. For example, since our work mainly deals with malicious attack, it can be considered to investigate an effect of cascading failures [25]. Link addition on modular networks for studying robustness [26] could be considered because SW property emerge by not only random rewiring but also link addition [27].

We obtain similar results for different attacks or values of *d* and *m*_*o*_ as follows. Figures from S1 to S8 Figs show the 1st LCC size against MB, IB attacks and RF in rewired networks with *d* = 4, 9 and 19. The 1st LCC size against MB attacks with *d* = 9 is already shown in Fig 2. S11–S13 Figs show the average path length in rewired networks with *d* = 4, 9 and 19 for varying *N*_*m*_ from 5, 10, and 20 as cliques to 100. Thus, for each *N*_*m*_, the network size *N* = *N*_*m*_ × *m*_*o*_ is changed by *m*_*o*_, while the minimum value of *m*_*o*_ is 3. S14 Fig show disparities of *L* between the values for rewired modular networks with *N*_*m*_ = 100 and theoretical value. S15 and S16 Figs show the 1st LCC size against IB attacks and RF to compare the results on anti-modularization and modified modularization. Note that the number *m*_*o*_ of modules becomes the maximum when it is equal to the size of clique *K*_5_, *K*_10_ or *K*_20_. Since network size *N* is 10^4^, the maximum values of *m*_*o*_ are 2000, 1000, and 500 for each of module sizes *N*_*m*_ = 5, 10 and 20.

## Supporting information

S1 Fig The 1st LCC size against IB attacks in rewired networks with *d* = 9 and the number *m*_*o*_ of modules. (A) *m*_*o*_ = 1000, (B) *m*_*o*_ = 500, (C) *m*_*o*_ = 100, (D) *m*_*o*_ = 50, (E) *m*_*o*_ = 20, and (F) *m*_*o*_ = 5. Color lines represent the rewiring rates *w*′ on anti-modularization.
(TIF)

S2 Fig The 1st LCC size against RF in rewired networks with *d* = 9 and the number *m*_*o*_ of modules. (A) *m*_*o*_ = 1000, (B) *m*_*o*_ = 500, (C) *m*_*o*_ = 100, (D) *m*_*o*_ = 50, (E) *m*_*o*_ = 20, and (F) *m*_*o*_ = 5. Color lines represent the rewiring rates *w*′ on anti-modularization.
(TIF)

S3 Fig The 1st LCC size against MB attacks in rewired networks with *d* = 4 and the number *m*_*o*_ of modules. (A) *m*_*o*_ = 2000, (B) *m*_*o*_ = 1000, (C) *m*_*o*_ = 500, (D) *m*_*o*_ = 100, (E) *m*_*o*_ = 20, and (F) *m*_*o*_ = 5. Color lines represent the rewiring rates *w*′ on anti-modularization.
(TIF)

S4 Fig The 1st LCC size against IB attacks in rewired networks with *d* = 4 and the number *m*_*o*_ of modules. (A) *m*_*o*_ = 2000, (B) *m*_*o*_ = 1000, (C) *m*_*o*_ = 500, (D) *m*_*o*_ = 100, (E) *m*_*o*_ = 20, and (F) *m*_*o*_ = 5. Color lines represent the rewiring rates *w*′ on anti-modularization.
(TIF)

S5 Fig The 1st LCC size against RF in rewired networks with *d* = 4 and the number *m*_*o*_ of modules. (A) *m*_*o*_ = 2000, (B) *m*_*o*_ = 1000, (C) *m*_*o*_ = 500, (D) *m*_*o*_ = 100, (E) *m*_*o*_ = 20, and (F) *m*_*o*_ = 5. Color lines represent the rewiring rates *w*′ on anti-modularization.
(TIF)

S6 Fig The 1st LCC size against MB attacks in rewired networks with *d* = 19 and the number *m*_*o*_ of modules. (A) *m*_*o*_ = 500, (B) *m*_*o*_ = 200, (C) *m*_*o*_ = 100, (D) *m*_*o*_ = 50, (E) *m*_*o*_ = 20, and (F) *m*_*o*_ = 5. Color lines represent the rewiring rates *w*′ on anti-modularization.
(TIF)

S7 Fig The 1st LCC size against IB attacks in rewired networks with *d* = 19 and the number *m*_*o*_ of modules. (A) *m*_*o*_ = 500, (B) *m*_*o*_ = 200, (C) *m*_*o*_ = 100, (D) *m*_*o*_ = 50, (E) *m*_*o*_ = 20, and (F) *m*_*o*_ = 5. Color lines represent the rewiring rates *w*′ on anti-modularization.
(TIF)

S8 Fig The 1st LCC size against RF in rewired networks with *d* = 19 and the number *m*_*o*_ of modules. (A) *m*_*o*_ = 500, (B) *m*_*o*_ = 200, (C) *m*_*o*_ = 100, (D) *m*_*o*_ = 50, (E) *m*_*o*_ = 20, and (F) *m*_*o*_ = 5. Color lines represent the rewiring rates *w*′ on anti-modularization.
(TIF)

S9 Fig Relation between modularity *Q* and six measures of robustness in rewired networks with *d* = 4. (A) Average betweenness centrality, (B) reciprocal of network efficiency, (C) average path length, (D) diameter, (E) spectral gap, (F) algebraic connectivity. Color lines represent the results for the numbers *m*_*o*_ of modules.
(TIF)

S10 Fig Relation between modularity *Q* and six measures of robustness in rewired networks with *d* = 19. (A) Average betweenness centrality, (B) reciprocal of network efficiency, (C) average path length, (D) diameter, (E) spectral gap, (F) algebraic connectivity. Color lines represent the results for the numbers *m*_*o*_ of modules.
(TIF)

S11 Fig Average path length *L* in rewired networks with *d* = 4 for varying the network size *N*. Rewired networks with (A) *N*_*m*_ = 5, (B) *N*_*m*_ = 20, (C) *N*_*m*_ = 50, and (D) *N*_*m*_ = 100. Color lines represent a rewiring rate *w*′ on anti-modularization.
(TIF)

S12 Fig Average path length *L* in rewired networks with *d* = 9 for varying the network size *N*. Rewired networks with (A) *N*_*m*_ = 10, (B) *N*_*m*_ = 50, and (C) *N*_*m*_ = 100. Color lines represent a rewiring rate *w*′ on anti-modularization.
(TIF)

S13 Fig Average path length *L* in rewired networks with *d* = 19 for varying the network size *N*. Rewired networks with (A) *N*_*m*_ = 20, (B) *N*_*m*_ = 100. Color lines represent a rewiring rate *w*′ on anti-modularization.
(TIF)

S14 Fig Difference of average path length *L* between the values for rewired modular networks and theoretical value. Rewired networks with (A) *d* = 4, *N*_*m*_ = 100, (B) *d* = 9, *N*_*m*_ = 100, and (C) *d* = 19, *N*_*m*_ = 100. Color lines represent a rewiring rate *w*′ on anti-modularization. Black solid and dashed lines indicate numerical and theoretical values for random *d*-regular graphs without modular structures.
(TIF)

S15 Fig Comparing the robustness against IB attacks in rewired networks on the anti-modularization and modified modularization. The 1st LCC size against MB attacks in rewired networks with *d* = 9 and the number *m*_*o*_ of modules. (A) *m*_*o*_ = 100, (B) *m*_*o*_ = 20, and (C) *m*_*o*_ = 5. Solid and dotted color lines represent the results by rewiring with *w*′ on anti-modularization and w on modified modularization.
(TIF)

S16 Fig Comparing the robustness against RF in rewired networks on the anti-modularization and modified modularization. The 1st LCC size against MB attacks in rewired networks with *d* = 9 and the number *m*_*o*_ of modules. (A) *m*_*o*_ = 100, (B) *m*_*o*_ = 20, and (C) *m*_*o*_ = 5. Solid and dotted color lines represent the results by rewiring with *w*′ on anti-modularization and w on modified modularization.
(TIF)
