# Supplementary figures and images for: Anti-modularization for both high robustness and efficiency including the optimal case
Source: PLoS One. 2024 Mar 28;19(3):e0301269. doi: 10.1371/journal.pone.0301269 (PMC10977745; doi:10.1371/journal.pone.0301269)

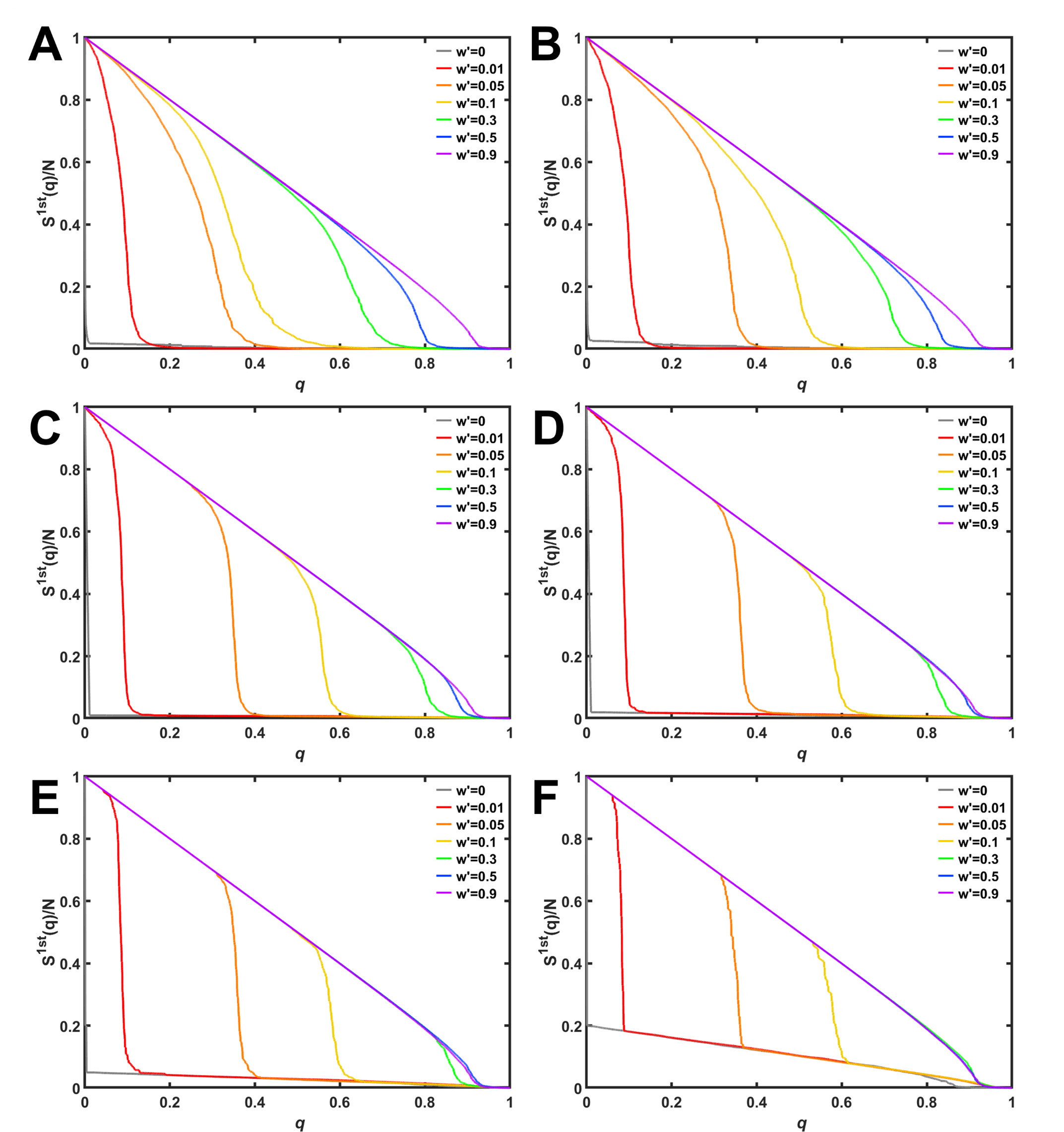

Supplement: S1 Fig — (A) mo = 1000, (B) mo = 500, (C) mo = 100, (D) mo = 50, (E) mo = 20, and (F) mo = 5. Color lines represent the rewiring rates w′ on anti-modularization. (TIF) [file pone.0301269.s001.tif]

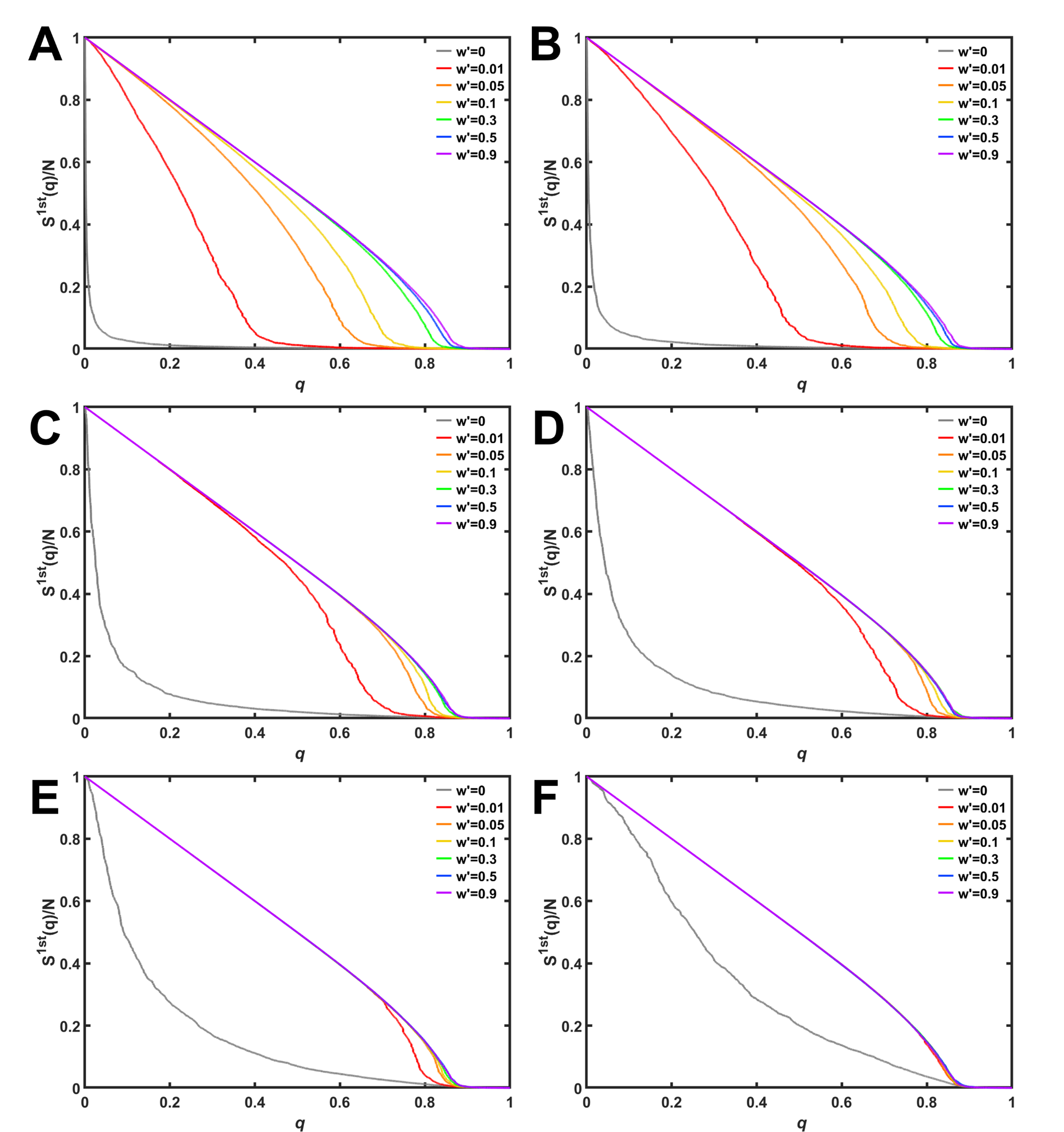

Supplement: S2 Fig — (A) mo = 1000, (B) mo = 500, (C) mo = 100, (D) mo = 50, (E) mo = 20, and (F) mo = 5. Color lines represent the rewiring rates w′ on anti-modularization. (TIF) [file pone.0301269.s002.tif]

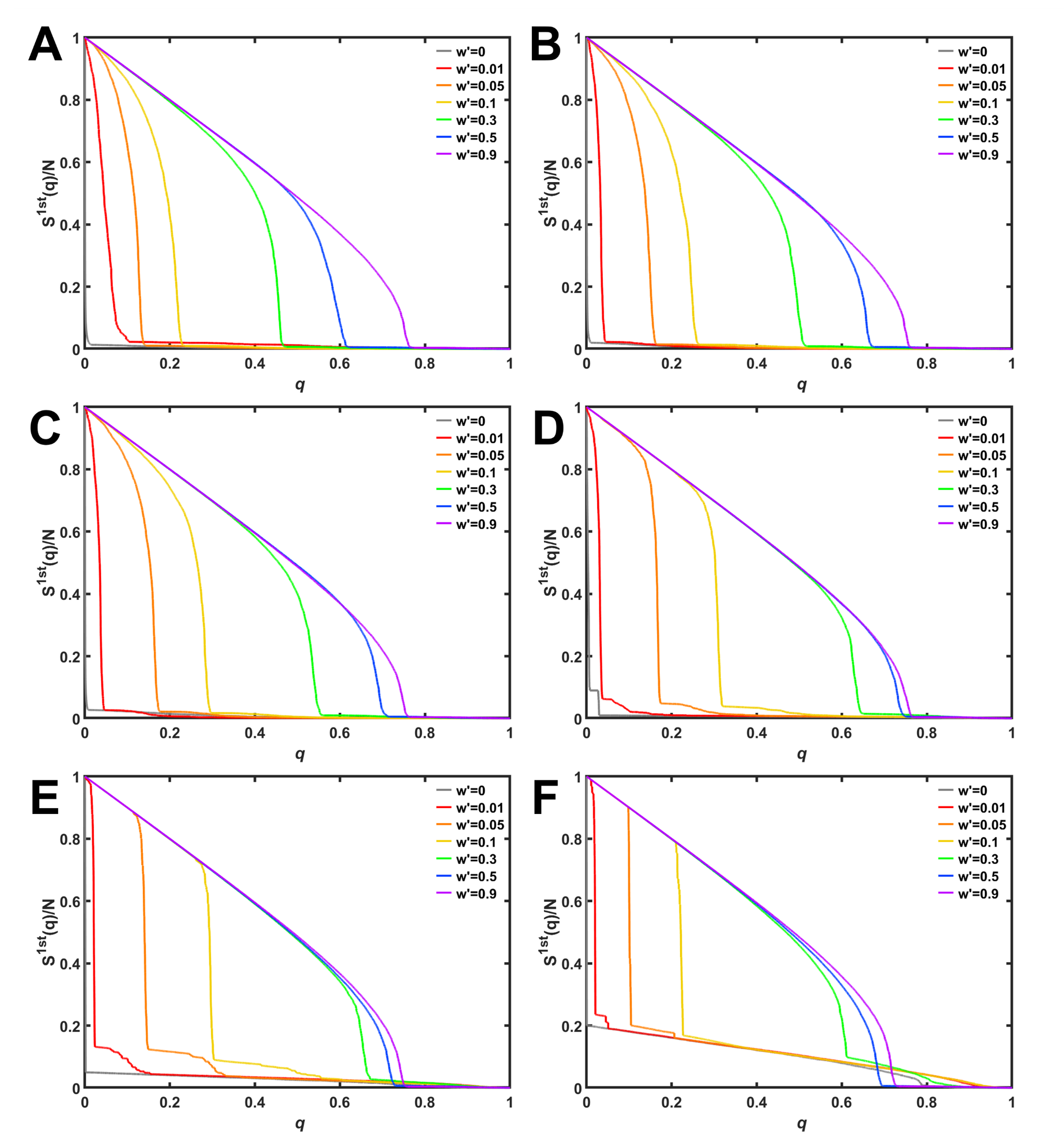

Supplement: S3 Fig — (A) mo = 2000, (B) mo = 1000, (C) mo = 500, (D) mo = 100, (E) mo = 20, and (F) mo = 5. Color lines represent the rewiring rates w′ on anti-modularization. (TIF) [file pone.0301269.s003.tif]

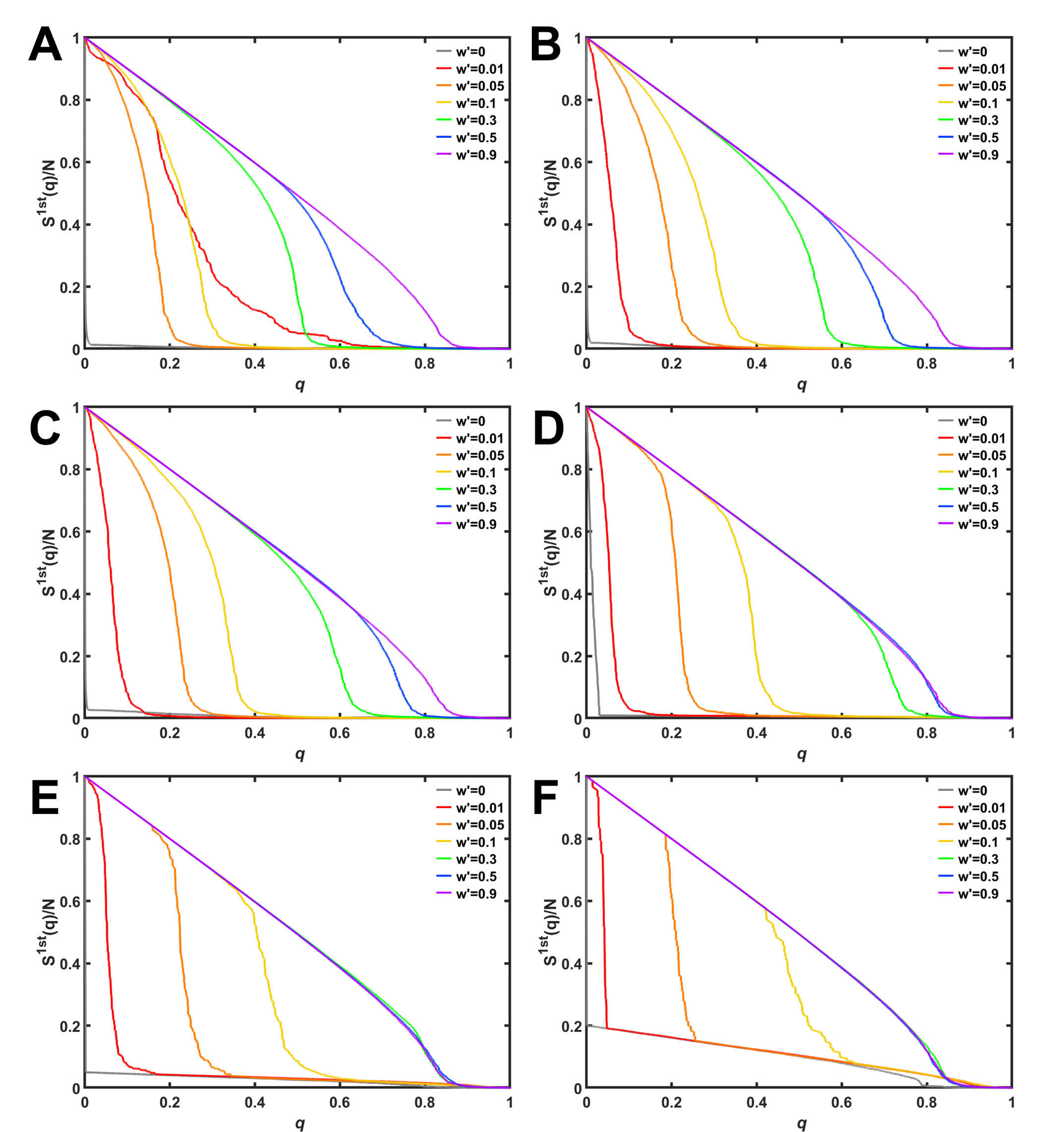

Supplement: S4 Fig — (A) mo = 2000, (B) mo = 1000, (C) mo = 500, (D) mo = 100, (E) mo = 20, and (F) mo = 5. Color lines represent the rewiring rates w′ on anti-modularization. (TIF) [file pone.0301269.s004.tif]

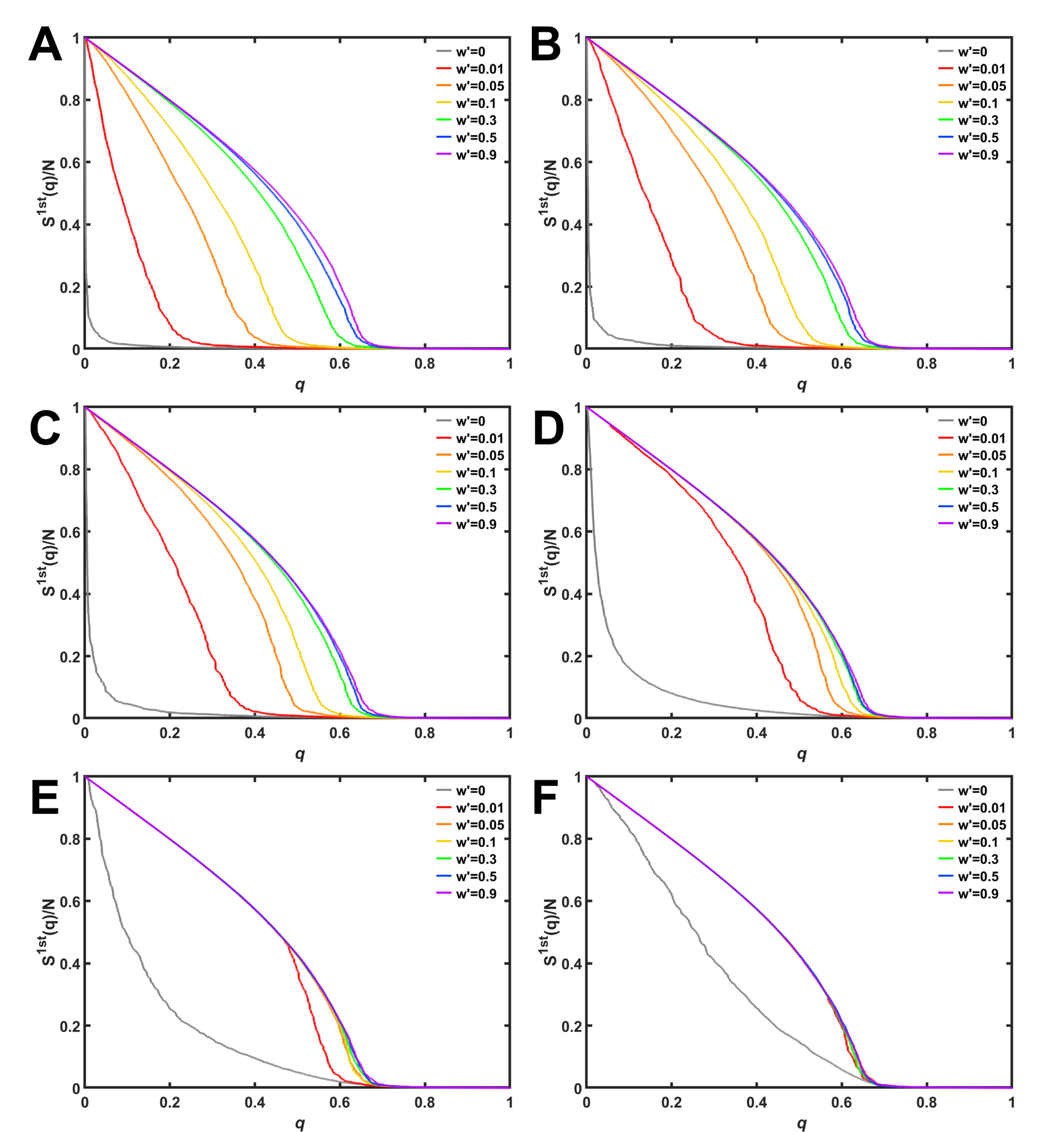

Supplement: S5 Fig — (A) mo = 2000, (B) mo = 1000, (C) mo = 500, (D) mo = 100, (E) mo = 20, and (F) mo = 5. Color lines represent the rewiring rates w′ on anti-modularization. (TIF) [file pone.0301269.s005.tif]

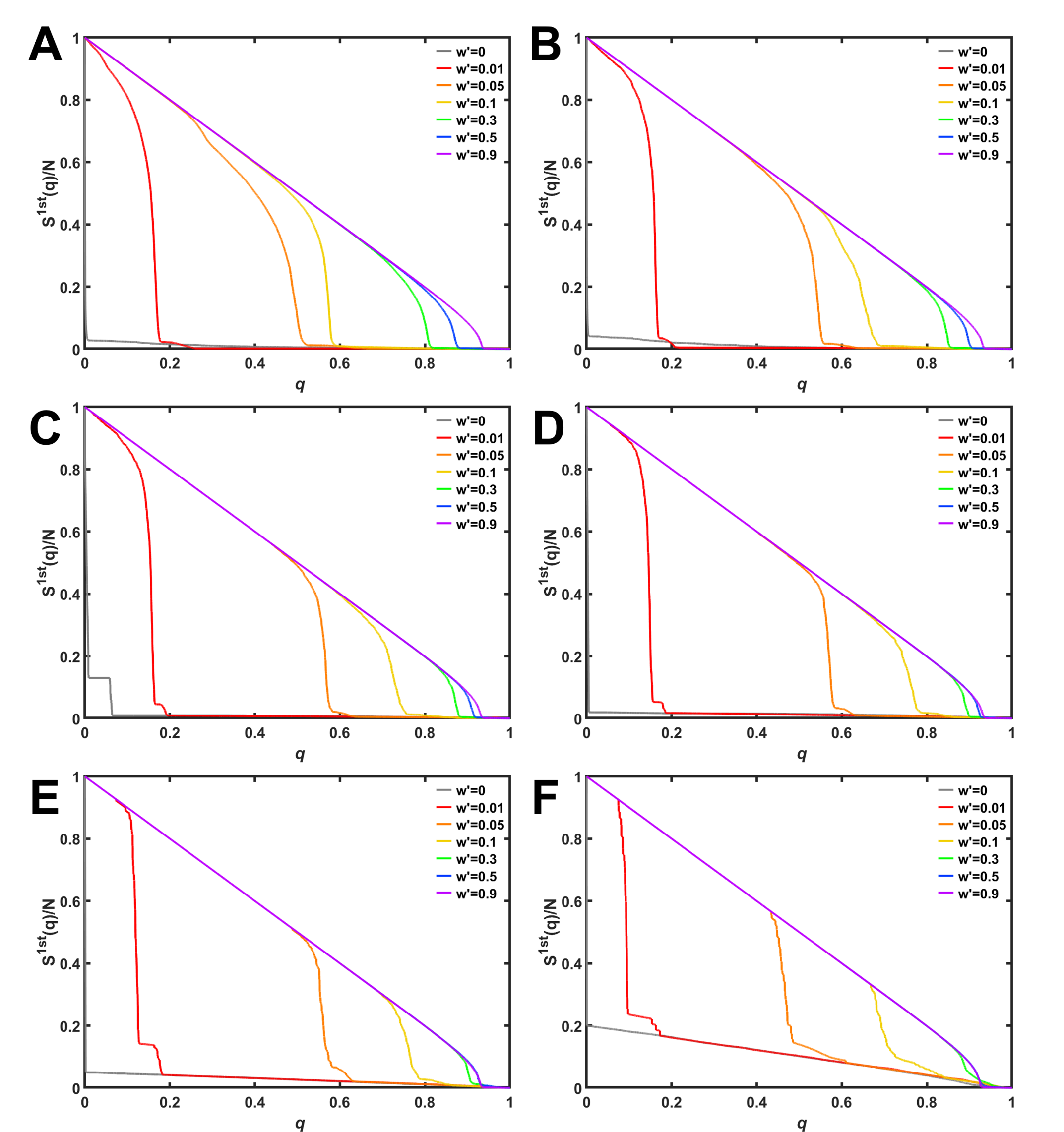

Supplement: S6 Fig — (A) mo = 500, (B) mo = 200, (C) mo = 100, (D) mo = 50, (E) mo = 20, and (F) mo = 5. Color lines represent the rewiring rates w′ on anti-modularization. (TIF) [file pone.0301269.s006.tif]

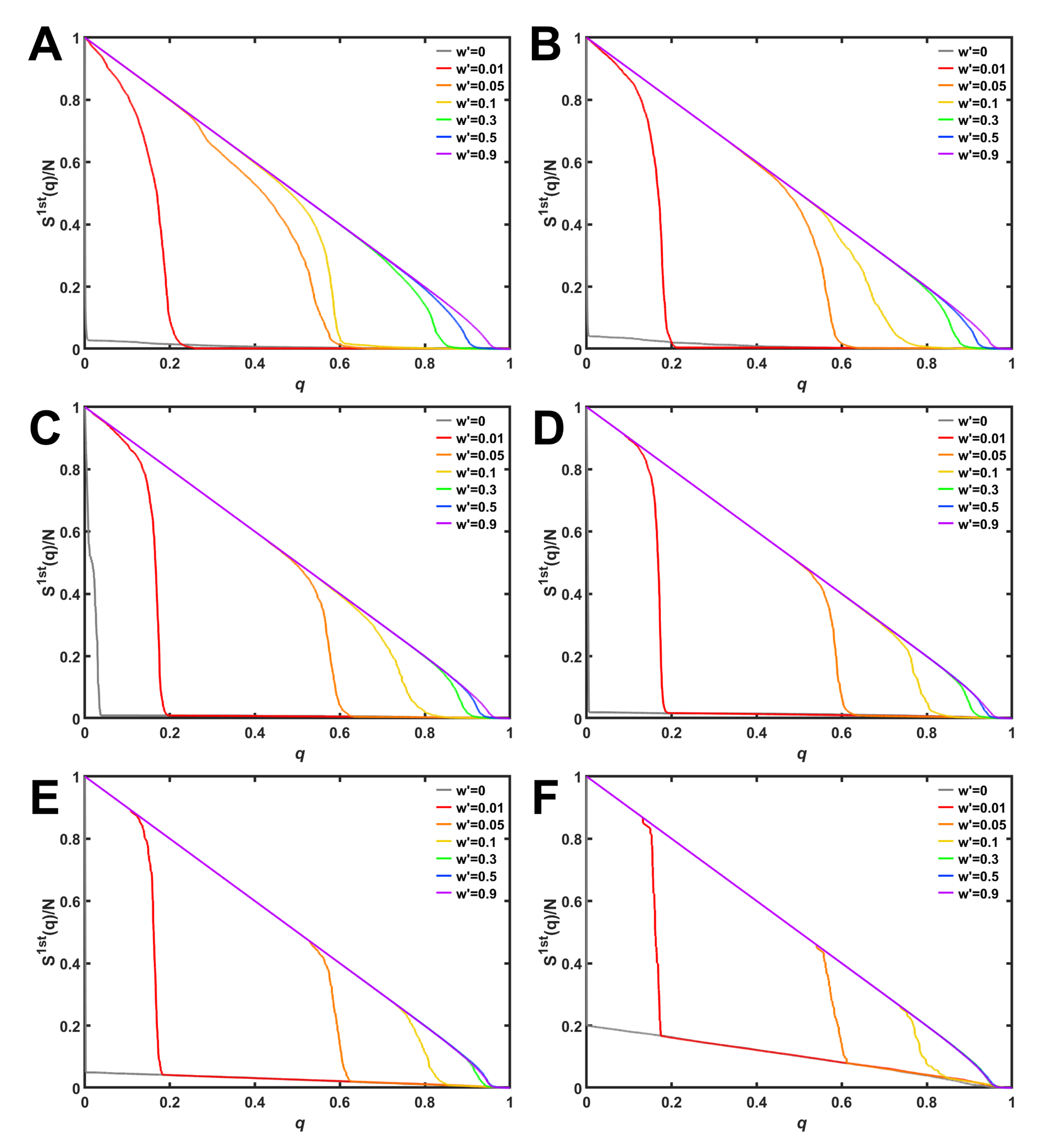

Supplement: S7 Fig — (A) mo = 500, (B) mo = 200, (C) mo = 100, (D) mo = 50, (E) mo = 20, and (F) mo = 5. Color lines represent the rewiring rates w′ on anti-modularization. (TIF) [file pone.0301269.s007.tif]

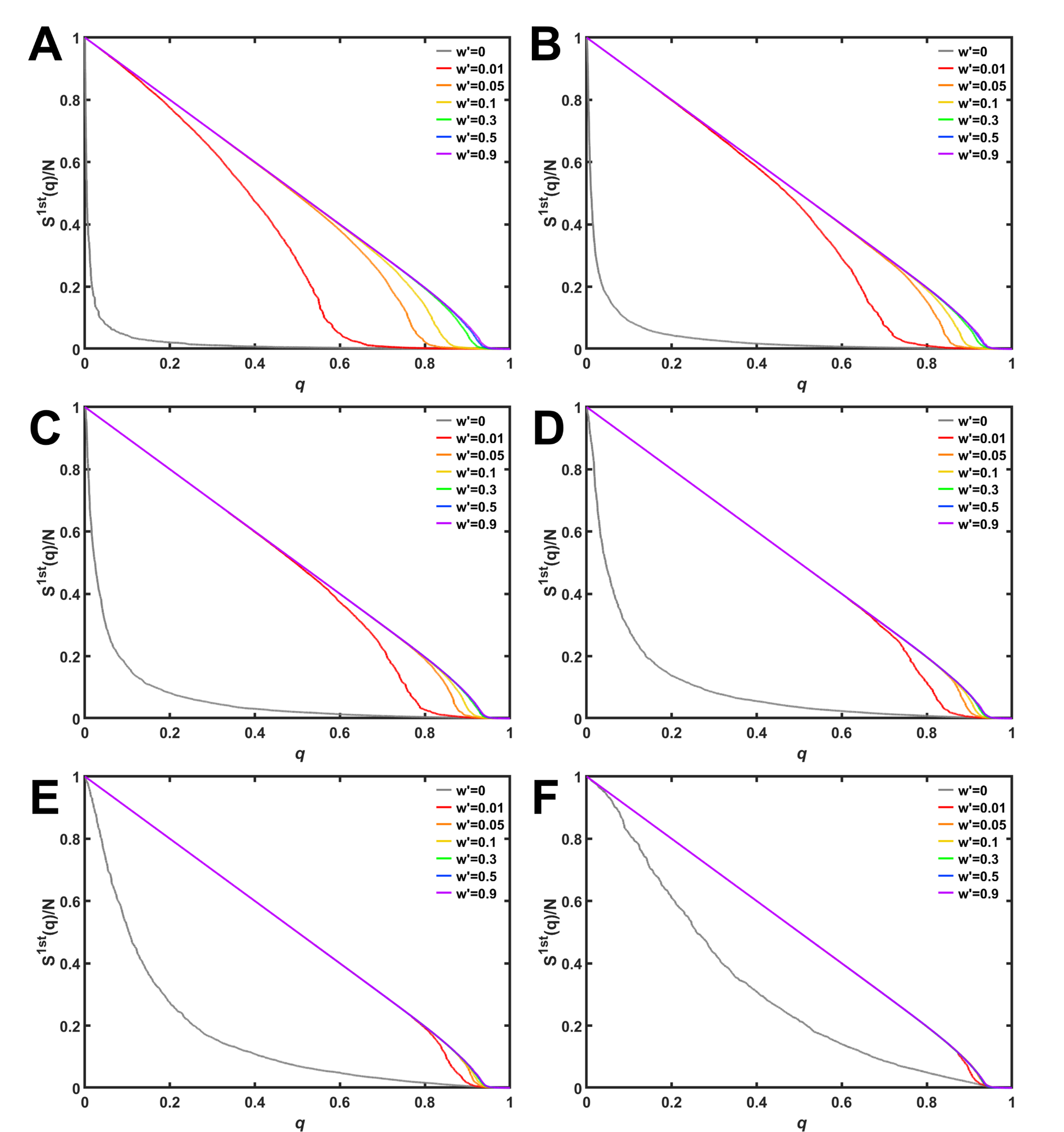

Supplement: S8 Fig — (A) mo = 500, (B) mo = 200, (C) mo = 100, (D) mo = 50, (E) mo = 20, and (F) mo = 5. Color lines represent the rewiring rates w′ on anti-modularization. (TIF) [file pone.0301269.s008.tif]

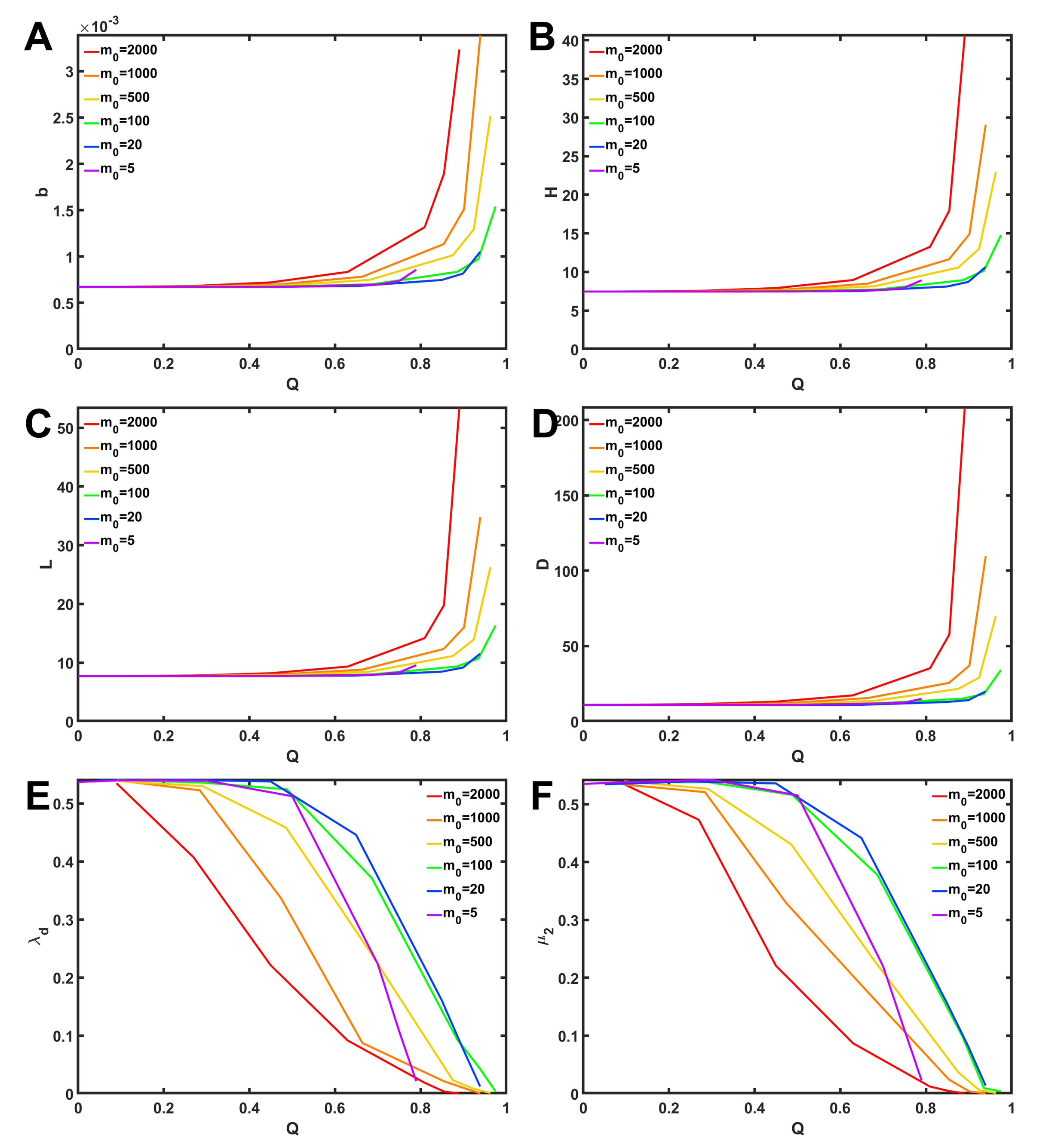

Supplement: S9 Fig — (A) Average betweenness centrality, (B) reciprocal of network efficiency, (C) average path length, (D) diameter, (E) spectral gap, (F) algebraic connectivity. Color lines represent the results for the numbers mo of modules. (TIF) [file pone.0301269.s009.tif]

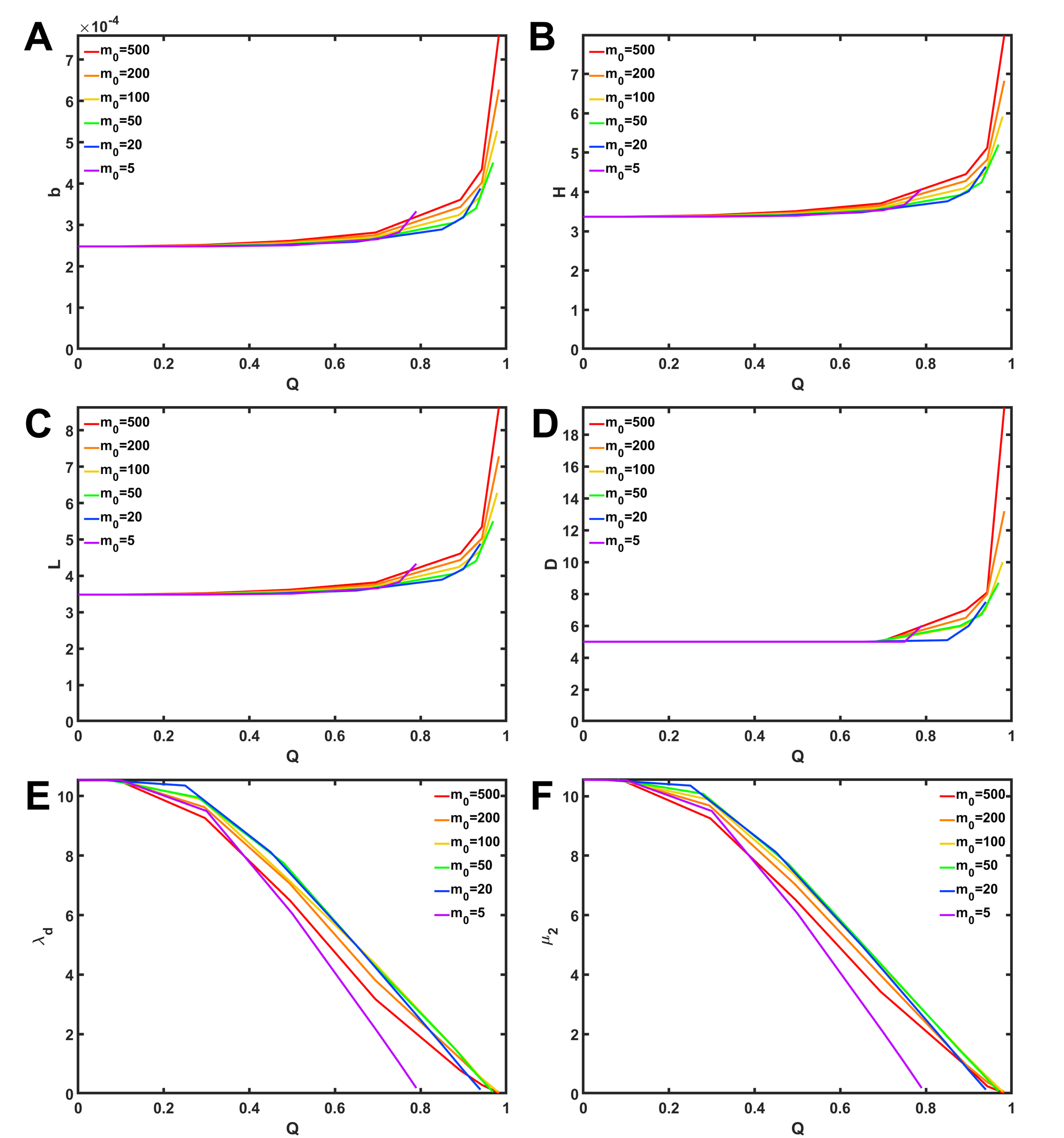

Supplement: S10 Fig — (A) Average betweenness centrality, (B) reciprocal of network efficiency, (C) average path length, (D) diameter, (E) spectral gap, (F) algebraic connectivity. Color lines represent the results for the numbers mo of modules. (TIF) [file pone.0301269.s010.tif]

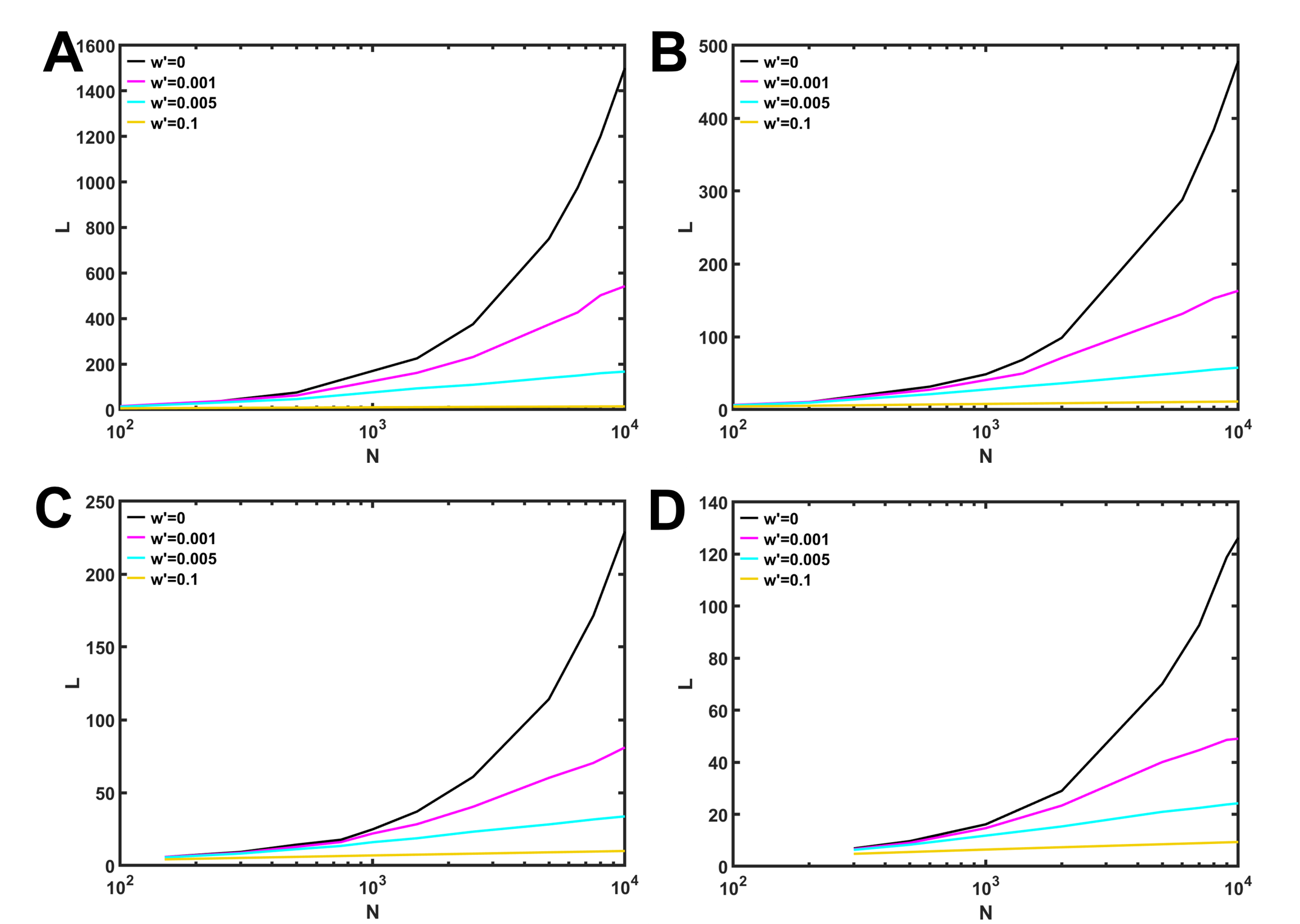

Supplement: S11 Fig — Rewired networks with (A) Nm = 5, (B) Nm = 20, (C) Nm = 50, and (D) Nm = 100. Color lines represent a rewiring rate w′ on anti-modularization. (TIF) [file pone.0301269.s011.tif]

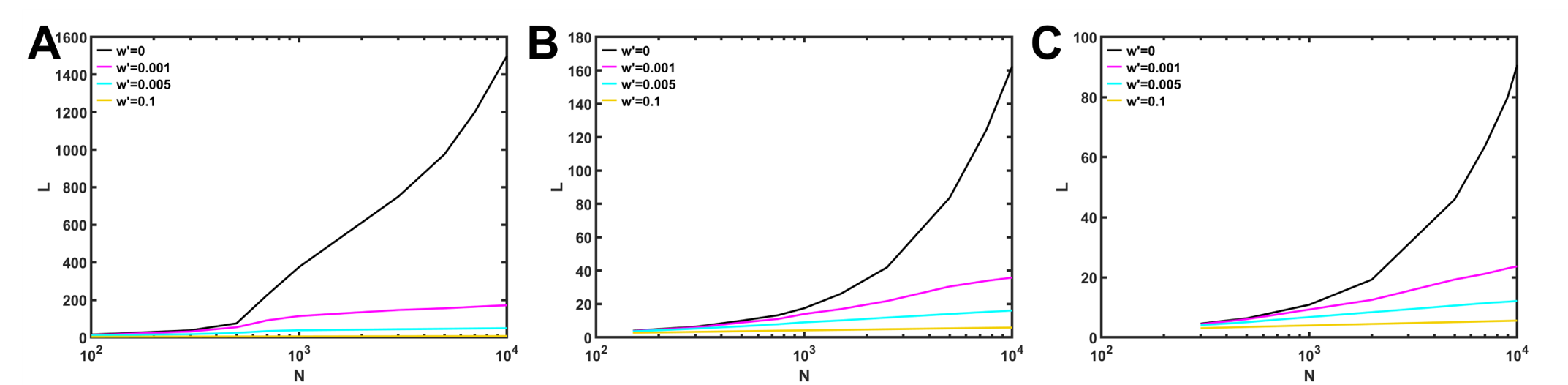

Supplement: S12 Fig — Rewired networks with (A) Nm = 10, (B) Nm = 50, and (C) Nm = 100. Color lines represent a rewiring rate w′ on anti-modularization. (TIF) [file pone.0301269.s012.tif]

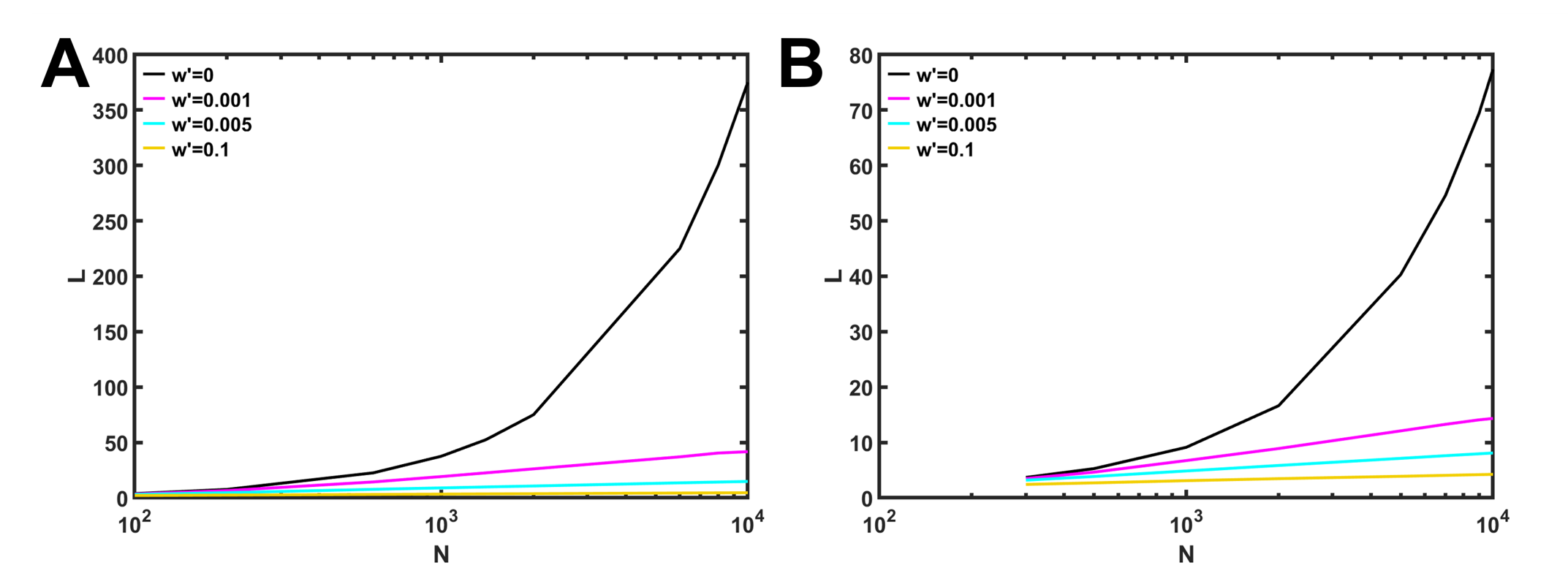

Supplement: S13 Fig — Rewired networks with (A) Nm = 20, (B) Nm = 100. Color lines represent a rewiring rate w′ on anti-modularization. (TIF) [file pone.0301269.s013.tif]

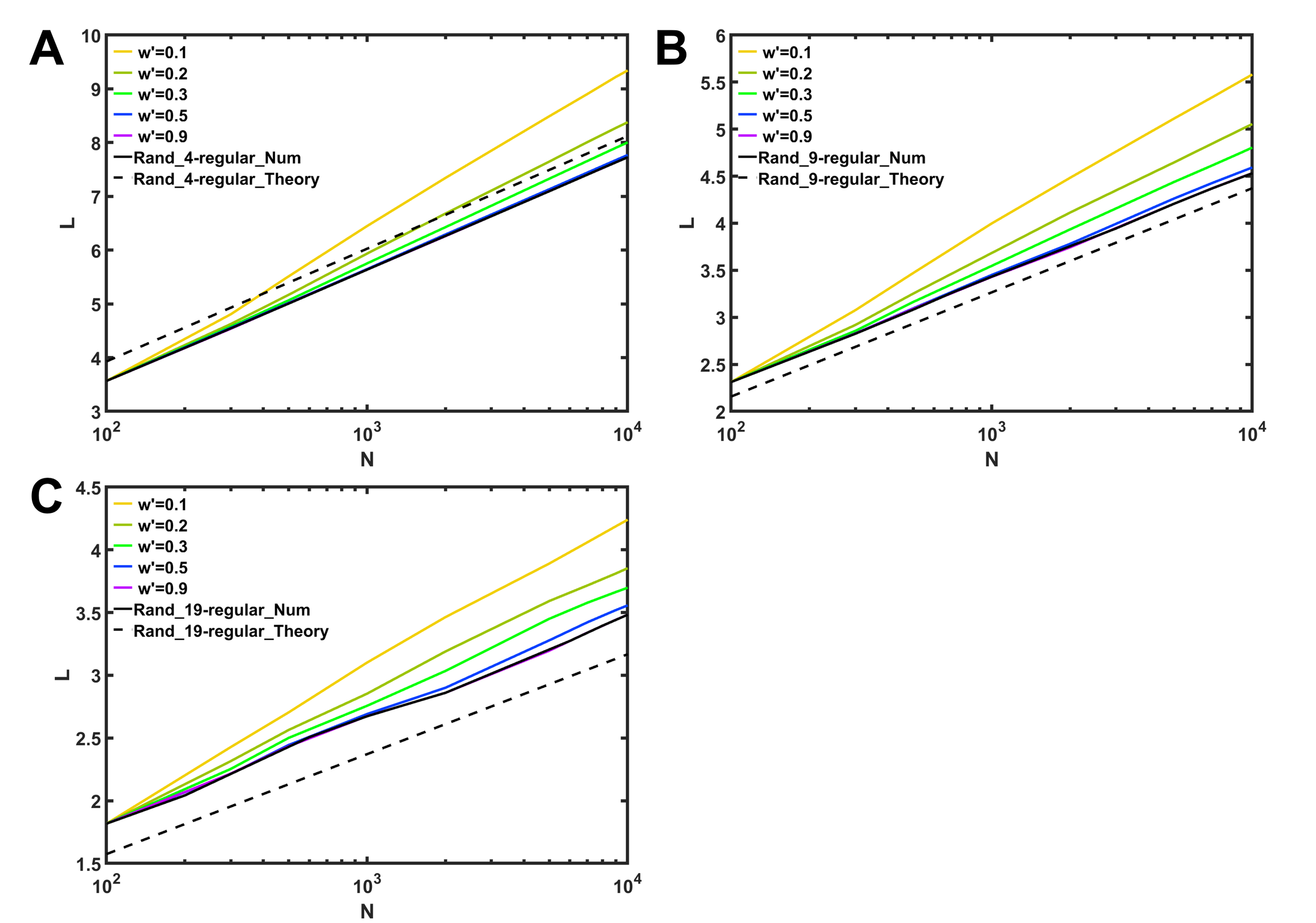

Supplement: S14 Fig — Rewired networks with (A) d = 4, Nm = 100, (B) d = 9, Nm = 100, and (C) d = 19, Nm = 100. Color lines represent a rewiring rate w′ on anti-modularization. Black solid and dashed lines indicate numerical and theoretical values for random d-regular graphs without modular structures. (TIF) [file pone.0301269.s014.tif]

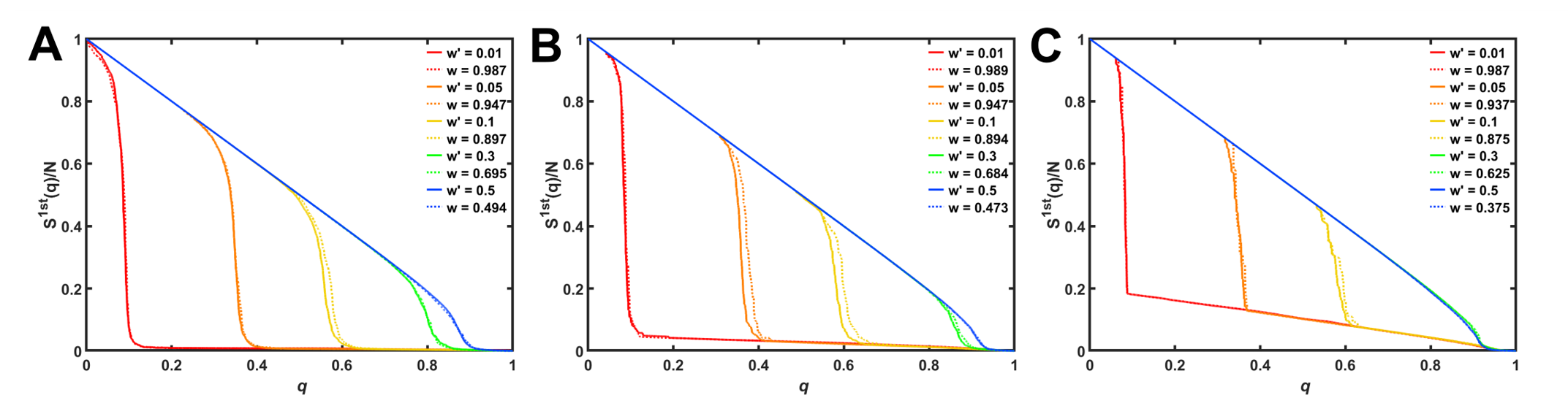

Supplement: S15 Fig — The 1st LCC size against MB attacks in rewired networks with d = 9 and the number mo of modules. (A) mo = 100, (B) mo = 20, and (C) mo = 5. Solid and dotted color lines represent the results by rewiring with w′ on anti-modularization and w on modified modularization. (TIF) [file pone.0301269.s015.tif]

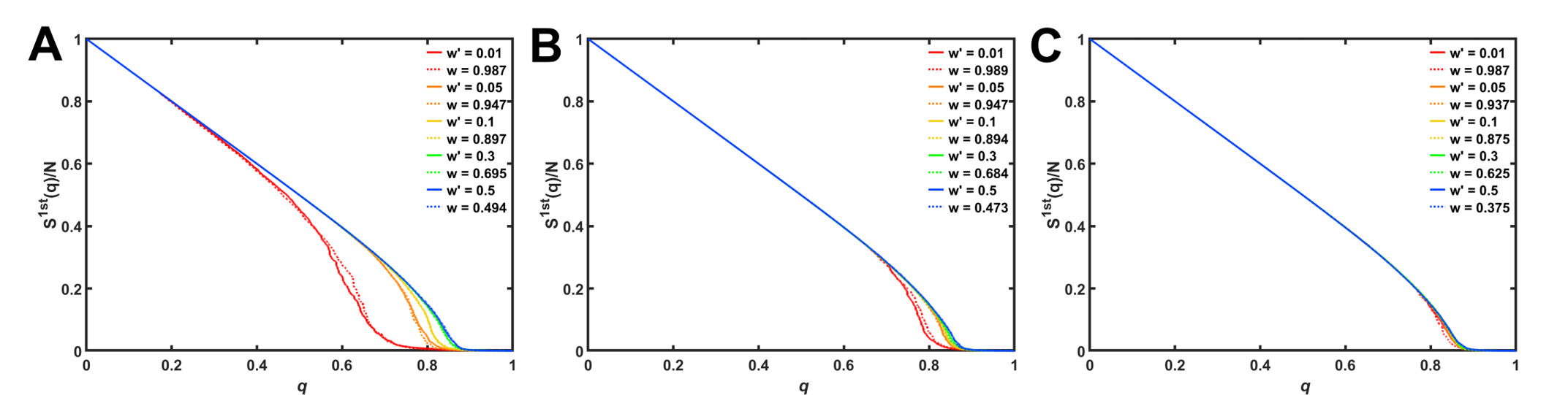

Supplement: S16 Fig — The 1st LCC size against MB attacks in rewired networks with d = 9 and the number mo of modules. (A) mo = 100, (B) mo = 20, and (C) mo = 5. Solid and dotted color lines represent the results by rewiring with w′ on anti-modularization and w on modified modularization. (TIF) [file pone.0301269.s016.tif]
